# Breast cancer patients suggestive of Li-Fraumeni syndrome: mutational spectrum, candidate genes, and unexplained heredity

**DOI:** 10.1186/s13058-018-1011-1

**Published:** 2018-08-07

**Authors:** Judith Penkert, Gunnar Schmidt, Winfried Hofmann, Stephanie Schubert, Maximilian Schieck, Bernd Auber, Tim Ripperger, Karl Hackmann, Marc Sturm, Holger Prokisch, Ursula Hille-Betz, Dorothea Mark, Thomas Illig, Brigitte Schlegelberger, Doris Steinemann

**Affiliations:** 1 Department of Human Genetics, Hannover Medical School, Carl-Neuberg-Strasse 1, 30625 Hannover, Germany; 2 Institute for Clinical Genetics, Faculty of Medicine Carl Gustav Carus, TU Dresden, Dresden, Germany; 3 German Cancer Research Center (DKFZ), Heidelberg, Germany; 4 National Center for Tumor Diseases (NCT) Partner Site Dresden, Dresden, Germany; 5 Institute of Medical Genetics and Applied Genomics, University of Tübingen, Tübingen, Germany; 6 Institute of Human Genetics, Helmholtz Zentrum München, Neuherberg, Germany; 7 Department of Gynecology and Obstetrics, Hannover Medical School, Hannover, Germany; 8 Department of Internal Medicine, Hematology/Oncology, University Hospital Frankfurt, Frankfurt, Germany

**Keywords:** Breast cancer, HBOC, Li-Fraumeni syndrome, Li-Fraumeni-like syndrome, *TP53*, Fanconi pathway, RECQ family, *CDKN2A*, *FANCA*

## Abstract

**Background:**

Breast cancer is the most prevalent tumor entity in Li-Fraumeni syndrome. Up to 80% of individuals with a Li-Fraumeni-like phenotype do not harbor detectable causative germline *TP53* variants. Yet, no systematic panel analyses for a wide range of cancer predisposition genes have been conducted on cohorts of women with breast cancer fulfilling Li-Fraumeni(-like) clinical diagnostic criteria.

**Methods:**

To specifically help explain the diagnostic gap of *TP53* wild-type Li-Fraumeni(-like) breast cancer cases, we performed array-based CGH (comparative genomic hybridization) and panel-based sequencing of 94 cancer predisposition genes on 83 breast cancer patients suggestive of Li-Fraumeni syndrome who had previously had negative test results for causative *BRCA1, BRCA2,* and *TP53* germline variants.

**Results:**

We identified 13 pathogenic or likely pathogenic germline variants in ten patients and in nine genes, including four copy number aberrations and nine single-nucleotide variants or small indels. Three patients presented as double-mutation carriers involving two different genes each. In five patients (5 of 83; 6% of cohort), we detected causative pathogenic variants in established hereditary breast cancer susceptibility genes (i.e., *PALB2, CHEK2, ATM*). Five further patients (5 of 83; 6% of cohort) were found to harbor pathogenic variants in genes lacking a firm association with breast cancer susceptibility to date (i.e., Fanconi pathway genes, RECQ family genes, *CDKN2A*/p14^ARF^, and *RUNX1*).

**Conclusions:**

Our study details the mutational spectrum in breast cancer patients suggestive of Li-Fraumeni syndrome and indicates the need for intensified research on monoallelic variants in Fanconi pathway and RECQ family genes. Notably, this study further reveals a large portion of still unexplained Li-Fraumeni(-like) cases, warranting comprehensive investigation of recently described candidate genes as well as noncoding regions of the *TP53* gene in patients with Li-Fraumeni(-like) syndrome lacking *TP53* variants in coding regions.

**Electronic supplementary material:**

The online version of this article (10.1186/s13058-018-1011-1) contains supplementary material, which is available to authorized users.

## Background

Li-Fraumeni syndrome (LFS) is a rare but highly penetrant cancer predisposition syndrome characterized by the early onset and familial aggregation of a variety of malignant neoplasms [1]. Germline pathogenic variants (PVs) in the tumor suppressor gene *TP53* are primarily responsible for this autosomal dominantly inherited disease [2]; however, because *TP53* PVs can be confirmed in only about 70% of suspected families [3], diagnosis of LFS is usually based on clinical evaluation and conformance to stringent criteria independent of mutational status. Different diagnostic criteria with varying stringency in terms of tumor abundance, age of onset, and spectrum of malignancies are in use, including classic LFS criteria, Birch’s and Eeles’ Li-Fraumeni-like syndrome (LFL) criteria, and several versions of the Chompret criteria [1, 4–9]. Although in principle any type of neoplasm may occur, a set of core cancers, namely breast cancer (BC), sarcomas, brain tumors, adrenocortical carcinomas, and leukemia, are expected to account for up to 77% of all tumor types occurring in patients with LFS [10].

Penetrance is remarkably high in carriers of germline *TP53* PVs, with 84% of female carriers and 41% of male carriers developing a tumor by age 45 years [11]. The gender difference is due to BC being one of the most predominating factors in this syndrome, representing up to 80% of all cancer cases in the age class of 16–45 years in females with LFS [10, 11]. However, because the syndrome itself is rare, the contribution of *TP53* germline alterations to hereditary BC overall is estimated to be less than 1% [12]. Because patients with BC harboring germline *TP53* PVs typically present with very early age of onset, routine *TP53* testing has been suggested for women who develop BC before the age of 30 years, independent of family history, and *TP53* detection rates within cohorts of patients with early-onset BC have been reported to be between 4% and 8% [8, 13, 14]. The 2008 and 2015 versions of the revised Chompret criteria [7, 9] are the sole criteria incorporating this important factor into their diagnostics, allowing for patients with early-onset BC (< 36 years) or very early-onset BC (< 31 years), respectively, to be included in LFS/LFL diagnostic procedures. Yet, two recent studies focused on cohorts of women meeting hereditary breast and ovarian cancer (HBOC) criteria clearly illustrate that a large percentage of germline *TP53* mutation carriers may still be missed by current criteria; the two studies reported *TP53* PVs in 13 patients overall, half of whom did not clinically meet either classic LFS or Chompret criteria, nor did they present with very early-onset disease [15, 16].

While not all germline *TP53* mutation carriers may be covered by LFS/LFL criteria, many families who do clinically conform to LFS/LFL criteria lack detectable germline *TP53* PVs, which is demonstrated by *TP53* mutation detection rates ranging from ~ 55% to 70% in classic LFS criteria, ~ 25% to 30% in LFL criteria, and ~ 20% to 35% in Chompret criteria [3, 6, 13]. This means that up to 45% of patients meeting classical LFS criteria and up to 80% of patients meeting Chompret or LFL criteria are left unexplained in a genetic sense. Few other candidate genes in the *TP53* pathway have been investigated in this context, one of them being *CDKN2A,* which was recently found to be mutated on a germline level in several LFL families in which the index case had a sarcoma [17]. Moreover, germline PVs in *CHEK2* have continuously but controversially been implicated in LFL phenotypes.

To the best of our knowledge, no systematic panel analyses for a wide range of known and suspected cancer predisposition genes have been conducted on cohorts of women with BC fulfilling LFL clinical diagnostic criteria. To further address this issue, as well as to elucidate the mutational spectrum in German population subjects with BC suggestive of LFS/LFL, we conducted both massive parallel sequencing and copy number analyses for a set of 94 cancer predisposition genes in a cohort of 83 *TP53*-negative and *BRCA1/2*-negative BC patients from the German population who met at least one of the hitherto suggested Li-Fraumeni-related criteria (LFS/LFL/Chompret criteria).

## Methods

### Study cohort

#### Patient selection

Eighty-three unrelated study subjects were selected from among a pool of female patients with BC who conformed to the German consortium criteria for HBOC [18] and whose pedigree had been established during genetic counseling in the tumor genetics outpatient clinic at Hannover Medical School, Germany, between 2002 and 2015. Prior to study inclusion, all patients had a negative test result for pathogenic single-nucleotide variants or small indels (Sanger sequencing) as well as gross genomic rearrangements (multiplex ligation-dependent probe amplification [MLPA], SALSA MLPA kits [MRC Holland, Amsterdam, The Netherlands], P002B/P087 for *BRCA1,* P045-B3/P077 for *BRCA2,* and P056-C1 for *TP53*) within the genes *BRCA1, BRCA2,* and *TP53*. Final inclusion of study participants was based on personal medical or family history characteristics suggestive of Li-Fraumeni-like traits and conformance to at least one of the hitherto existing Li-Fraumeni-related criteria (LFS/LFL/Chompret criteria). Classification into LFS/LFL and Chompret criteria was performed either on the particular proband undergoing mutation analysis or on another index case within the same family. The majority of patients were assumed to be of European-Caucasian ancestry. All patients signed informed consent forms, and the project was approved by the research ethics committee of Hannover Medical School (approval number 3528).

#### Cohort characteristics

Among our cohort of 83 patients with BC, 50 unrelated women had early-onset BC (age at diagnosis ranging from 19 to 34 years, median age 29 years), 9 index cases were diagnosed with bilateral BC or two primary independent breast carcinomas, 4 individuals harbored at least one independent neoplasm besides BC, in 10 families either the index patient or one of her relatives had a sarcoma, and in 16 families either the index patient or a relative was diagnosed with a brain tumor (multiple entries being possible).

### Next-generation sequencing and bioinformatics analysis

Germline DNA was isolated from peripheral blood leukocytes according to standard procedures. For library preparation, the Illumina TruSight Cancer Panel (Illumina, San Diego, CA, USA) was used (*see* Additional file 1 for the entire list of the 94 included genes and SNPs). All samples were processed according to the manufacturer’s recommendations and sequenced on an Illumina NextSeq 500 platform. Mean sequence depth was at least 100 × for each sample. A minimum sequence depth of 20 × for at least 97% of the region of interest (ROI) (i.e., coding regions and the first two base pairs [bp] of flanking intronic regions) could be obtained for 81 of 83 samples; 2 samples failed to confer to these quality parameters. The majority of samples (68 of 83; 82%) reached 20 × coverage for at least 99% of the target region. All 94 panel genes were analyzed using the megSAP analysis pipeline [19], filtering for variants with a minimum variant allele frequency (VAF) of 15%, a relatively low VAF that was chosen for the eventuality of constitutional *TP53* mosaicism [20]. Yet, all detected PVs exhibited a VAF of > 40% (range, 43–56%). Reported nucleotide positions refer to GRCh37/hg19.

Integrative Genomics Viewer (IGV) [21, 22] was employed to visualize sequencing results. Variants were further filtered with the software GSvar (part of ngs-bits [23]) to identify frameshift, nonsense, splice site, missense, inframe insertion/deletion, and 3′/5′ untranslated region (UTR) variants that had a minor allele frequency (MAF) at or below 0.1% in the 1000 Genomes Project [24], ExAC [25], and Kaviar [26] databases and were located in the ROI as defined above. As a second measure, via GSvar analysis, we filtered all variants that were predicted to be pathogenic by at least 2 of 4 in silico prediction tools (i.e., MetaLR [27], Sift [28], PolyPhen-2 HVAR, and PolyPhen-2 HDIV [29]) or that were documented as pathogenic in either the ClinVar [30] or HGMD [31] database, as long as they were neither listed *n* > 2 in the FLOSSIES database of healthy older women [32], nor classified as class 1–2 variants according to a consented expert decision within the German HBOC Consortium, nor reported as predominantly benign or likely benign in ClinVar [30]. Variants were classified according to American College of Medical Genetics and Genomics (ACMG) guidelines [33]. For splicing prediction, in silico splicing tools (i.e., SSF, MaxEnt, NNSplice, GeneSplicer, and HSF) included in Alamut software version 2.8 rev. 1 (interactive biosoftware, Rouen, France) were used. In addition, and with particular relevance to potential *TP53* variants of reduced penetrance or hypomorphic alleles, all variants detected within the *TP53* gene (including synonymous and intronic variants) were analyzed. Regarding *TP53*’s UTRs, the Illumina gene panel used captures approximately 70 bp of both 5′ and 3′ UTRs with adequate depth, resulting in only a small fraction of the 3′ UTR being covered.

### Array CGH and copy number evaluation

For detection of copy number changes, a custom-made eArray covering the identical set of genes targeted by Illumina’s TruSight Cancer Panel was used (SureDesign 069100, 8x60K; Agilent Technologies, Santa Clara, CA, USA) [34]. All samples were processed according to the manufacturer’s instructions. Microarray slides were scanned using an Agilent microarray scanner system, and standard settings of the Feature Extraction Software (version 11.0.1.1) were applied for data normalization. Data analysis was subsequently performed via Agilent’s Genomic Workbench (version 7.0.4.0). Nucleotide positions refer to GRCh37/hg19. Annotation of alterations was computed under different settings dependent on data quality. Verification of detected copy number variations with a second independent method was performed either via MLPA, if probe sets were commercially available for the relevant region (SALSA MLPA kits [MRC Holland], P042-B1 for *ATM*; P056 for *CHEK2*; P008-C1 for *PMS2*), and/or via next-generation sequencing (NGS)-based copy number evaluation using CnvHunter, which is part of ngs-bits [23].

## Results

### LFS/LFL classification details and performance of classification systems

Of the 83 families in our cohort, 48 met Eeles’ LFL criteria [5]; 23 met the original stringent Chompret criteria [6]; 75 were consistent with the temporarily suggested 2008 version of Chompret criteria, which loosened age restrictions and included all *BRCA1/2*-negative patients with BC before age 36 years, regardless of family history [7]; 43 met the 2009 version of Chompret criteria, which again reversed the latter point [8]; 53 met the current Chompret 2015 version criteria, which by definition include all *BRCA1/2*-negative patients with BC before age 31 years, regardless of family history [9]; 12 families met Birch’s LFL criteria [4]; and 1 family met classic LFS criteria [1] (multiple entries being possible). Considering mutation carriers’ families only (10 of 83 families), all 10 families were consistent with the 2008 version of Chompret criteria, whereas 6 of 10 could be classified into Eeles’ LFL and Chompret’s 2009 version criteria. When we examined the mutation detection rate for each classification system separately, we found that the majority of the applied criteria performed similarly, with the highest detection rate observed within members of both Eeles’ LFL criteria and Chompret’s original/2008/2009 criteria (13–14%).

For an overview of the classification results and phenotypic characteristics of the cohort, *see* Table 1. Information about LFS and LFL criteria definitions is given in Additional file 2.

**Table 1:** Classification and cohort characteristics

	Total sample, *n* (%)	Mutation carriers, *n* (%)	Mutation carriers per group, *n* (%)
Eeles’ LFL criteria	48/83 (58%)	6/10 (60%)	6/48 (13%)
Birch’s LFL criteria	12/83 (15%)	1/10 (10%)	1/12 (8%)
Original Chompret criteria	23/83 (28%)	3/10 (30%)	3/23 (13%)
Chompret 2008 version criteria	75/83 (90%)	10/10 (100%)	10/75 (13%)
Chompret 2009 version criteria	43/83 (52%)	6/10 (60%)	6/43 (14%)
Chompret 2015 version criteria	53/83 (64%)	5/10 (50%)	5/53 (9%)
Classic LFS criteria	1/83 (1%)	0/10	0/1
Early-onset BC (i.e., ≤ 34 years)	50/83 (60%)	7/10 (70%)	7/50 (14%)
Bilateral BC/two primary BCs	9/83 (11%)	2/10 (20%)	2/9 (22%)
Additional neoplasms besides BC	4/83 (5%)	0/10	0/4
Sarcoma in family or self	10/83 (12%)	0/10	0/10
Brain tumor in family or self	16/83 (20%)	2/10 (20%)	2/16 (13%)

*Abbreviations: BC* Breast cancer, *LFL* Li-Fraumeni-like syndrome, *LFS* Li-Fraumeni syndrome

*See* Additional file 2 regarding LFS/LFL criteria

### Variant detection in patients with LFL personal or family history

Within the cohort of 83 subjects with BC, we detected 13 pathogenic or likely pathogenic heterozygous germline variants (ACMG class 4–5; i.e., nonsense, frameshift, missense, [consensus] splice site variants, or copy number aberrations) in 10 unrelated patients and 9 genes [*ATM* (*n* = 3), *CDKN2A* (*n* = 1), *CHEK2* (*n* = 1), *FANCI* (*n* = 1), *PALB2* (*n* = 2), *PMS2* (*n* = 1), *RECQL4* (*n* = 2), *RUNX1* (*n* = 1), and *WRN* (*n* = 1)]. These 13 deleterious variants include 4 gross genomic deletions/insertions and 9 truncating NGS-detected variants. Altogether, 3 patients presented as double-mutation carriers, harboring 2 (likely) PVs each (i.e., *PMS2* and *FANCI, CDKN2A* and *RECQL4,* as well as *ATM* and *CHEK2*). Half of the 10 mutation carriers (5 of 83; 6% of the entire cohort) were found to harbor PVs in widely accepted BC susceptibility genes (i.e., *PALB2, ATM,* and *CHEK2*), whereas the remaining half (5 of 83; 6% of the entire cohort) carried PVs in candidate genes for which no firm association with BC incidence has yet been established in a heterozygous germline setting (*CDKN2A, RUNX1, FANCI, WRN,* and *RECQL4*). Four variants detected via NGS and 2 of the 4 copy number aberrations detected via array-based CGH (comparative genomic hybridization) have not previously been described in the literature. For an overview of all identified classes 4 and 5 variants, *see* Table 2. An overview of all index patients carrying (likely) pathogenic germline variants, including their personal and family histories as well as available clinical data and LFS/LFL classification, is given in Table 3, and respective pedigrees can be accessed in Additional file 3.

**Table 2:** Detected pathogenic and likely pathogenic single-nucleotide variants, small indels, and copy number alterations

Index patient identifier	Gene (transcript GRCh37/hg19)	Variant type	dbSNP	Exons/total no. of exons	cDNA change	Predicted amino acid change	Aberration array CGH	ACMG class	Previously reported^a^
7	*PALB2* (NM_024675.3)	Frameshift	rs515726124	4/13	c.509_510delGA	p.(Arg170Ilefs*14)		5	Yes
30	*RUNX1* (NM_001754.4)	Splice donor	rs375131372	3/8	c.97+1G>A			4	No
32	*ATM* (NM_000051.3)	Deletion		62–63/63			arr [GRCh37]11q22.3 (108233779_108240057)×1	5	Yes
40	*ATM* (NM_000051.3)	Nonsense		61/63	c.8793T>A	p.(Cys2931*)		5	Yes
	*CHEK2* (NM_007194.3)	Deletion		9–10/15			arr [GRCh37]22q12.1 (29092709_29097723)×1	5	Yes
58	*CDKN2A* (NM_058195.3)	Nonsense		2/3	c.292C>T	p.(Arg98*)		4	No
	*RECQL4* (NM_004260.3)	Splice donor		7/21	c.1390+1G>C			4	No
59	*WRN* (NM_000553.4)	Deletion		15–16/35			arr [GRCh37]8p12 (30948138_30949422)×1	4	No
60	*ATM* (NM_000051.3)	Nonsense	rs587779852	40/63	c.5932G>T	p.(Glu1978*)		5	Yes
65	*FANCI* (NM_001113378.1)	Nonsense	rs121918164	37/38	c.3853C>T	p.(Arg1285*)		5	Yes
	*PMS2* (NM_000535.5)	Deletion		3–8/15			arr [GRCh37]7p22.1 (6035238_6042593)×1	5	No
76	*PALB2* (NM_024675.3)	Frameshift	rs180177143	3/13	c.172_175delTTGT	p.(Gln60Argfs*7)		5	Yes
79	*RECQL4* (NM_004260.3)	Missense and splice region	rs186739072	16/22	c.2755G>A	p.(Ala919Thr)		4	No

*Abbreviations: ACMG* American College of Medical Genetics and Genomics, *cDNA* Complementary DNA, *CGH* Comparative genomic hybridization, *dbSNP* Single Nucleotide Polymorphism database

^a^Based on reports in the literature, ClinVar, Decipher, and gene-specific databases

**Table 3:** Clinical information and summary of personal and family histories of mutation carriers

Index patient identifier	Variant	Personal history (age at diagnosis in years)	Immunohistochemistry (if available)	Family history of cancer (age at diagnosis in years)	Conformance to LFS/LFL criteria^a^
7	PALB2:p.(Arg170Ilefs*14)	BC (40)	1. TNBC	Mat. – M: BC (40)	Eeles
				Pat. – F: leukemia (< 50), GM: esophagus (60)	Chompret 2008
					Chompret 2009
30	RUNX1:c.97+1G>A	BC (33)	N/A	Mat. – M: BC (56), GM: BC (60)	Chompret 2008
32	*ATM* exon 62–63 del	BC bilateral (30 + 40)	1. Triple-positive 2. Triple-positive	Mat. – M: OvCa (51), half-S: BC (41) + lung (46), U: leukemia (45), GM: cancer, GF: cancer	Eeles
					orig. Chompret
					Chompret 2008
					Chompret 2009
					Chompret 2015
40	ATM:p.(Cys2931*)	BC (39)	N/A	Mat. – M: OvCa (40), A: leukemia (20)	Eeles
	*CHEK2* exon 9–10 del				Chompret 2008
					Chompret 2009
58	CDKN2A*:*p.(Arg98*)	BC (32)	HER2+	Mat. – GM: ureter (64)	Chompret 2008
	RECQL4*:*c.1390+1G>C			Pat. – GF: hypopharynx (62)	
59	*WRN* exon 15–16 del	BC (32)	N/A	Mat. – half-S: melanoma (46), GF: lung (64), GM: CRC (50), GGM: CRC (59), U: kidney (60), this U’s sons: melanoma (45), basalioma (36)	Eeles
					orig. Chompret
					Chompret 2008
				Pat. – U: brain (25), 10 Us/As: all died of cancer at a young age	Chompret 2009
					Chompret 2015
					Birch
60	ATM:p.(Glu1978*)	BC (40)	HR+	Mat. – M: BC (51), GM: BC (73)	Eeles
				Pat. – F: glioblastoma (42)	Chompret 2008
					Chompret 2009
					Chompret 2015
65	FANCI:p.(Arg1285*)	BC (30)	HER2+	Mat. – U: CRC (37), GF: CRC (70), GGM: cancer	Chompret 2008
	*PMS2* exon 3–8 del			Pat. – GF: esophagus (74)	Chompret 2015
76	PALB2:p.(Gln60Argfs*7)	BC bilateral (33 + 39)	1. HR+ lobular 2. HR+ (HER2+ in metastases)	B: lung (43)	Eeles
				Mat. – M: pancreas (58), A: melanoma (67)	orig. Chompret
					Chompret 2008
					Chompret 2009
79	RECQL4:p.(Ala919Thr)	BC (27)	HR+	Mat. – M: NHL (42), GF: bladder (54)	Chompret 2008
				Pat. – GM: BC (53), GU: lymphoma (52), GA: BC (57), this GA’s daughter: BC (47)	Chompret 2015

*Abbreviations: A* Aunt, *B* Brother, *BC* Breast cancer, *CRC* Colorectal cancer, *del* Deletion, *F* Father, *GA* Grand aunt, *GF* Grandfather, *GGM* Great grandmother, *GM* Grandmother, *GU* Grand uncle, *HER2+ HER2* (*ERBB2*) overexpression/amplification, *HR+* Hormone receptor-positive, *M* Mother, *Mat.* Maternal, *N/A* Not accessible, *Ne* Nephew, *NHL* Non-Hodgkin lymphoma, *Ni* Niece, *OvCa* Ovarian cancer, *Pat.* Paternal, *S* Sister, *TNBC* Triple-negative breast cancer, *U* Uncle

^a^Information about Li-Fraumeni and Li-Fraumeni-like criteria definition are given in Additional file 2

Besides classes 4–5 variants, we detected 49 variants of unknown significance (VUS, ACMG class 3) within our collective for the parameters detailed above. Via array-based CGH, we identified a duplication of exons 1–21 of the *BLM* gene, which currently ranks as a class 3 variant owing to a lack of more precise breakpoint information. Via NGS-based sequencing, we detected 46 missense or disruptive inframe deletion and 2 splice region VUS with an MAF ≤ 0.1% occurring in 35 of the 94 investigated genes. Among this list of VUS, we observed an unexpected frequency of very rare *FANCA* missense variants that were linked to either very early-onset BC or exceptionally Li-Fraumeni-suggestive family/personal history. For a summary of all VUS with an MAF ≤ 0.1% detected via NGS-based sequencing, *see* Table 4. Neither of the identified VUS was included in the statistical analysis of this work.

**Table 4:** Missense and splice variants of unknown significance detected by NGS-based sequencing

Gene (transcript GRCh37/hg19)	Index patient identifier	Variant type	Exons/total no. of exons	cDNA change	Predicted amino acid change	dbSNP	Total allele frequency, gnomAD; population with highest AF^a^	In silico prediction^b^	Splicing predictions at nearest natural junction
*ALK* (NM_004304.4)	52	Splice region and synonymous	17/29	c.2817C>T	p.(Gly939Gly)	rs112022466	0.0001840; SA: 0.0006173		Native acceptor site: MaxEnt: + 23.7% NNSPLICE: + 9.5% HSF: + 0.9% GeneSplicer: + 32.0%
*APC* (NM_001127510.2)	83	Missense	17/17	c.4918C>T	p.(Arg1640Trp)	rs373440614	0.00007223;	C	
							O: 0.0001548		
*ATM* (NM_000051.3)	20	Missense	10/63	c.1271C>A	p.(Pro424His)	rs147472613	0.00002188;	D	
							A: 0.00004189		
*ATM* (NM_000051.3)	20	Missense	50/63	c.7357C>T	p.(Arg2453Cys)	rs755418571	0.00001219;	C	
							EA: 0.00005807		
*BRCA2* (NM_000059.3)	36	Missense	10/27	c.831T>G	p.(Asn277Lys)	rs28897705	0.00006632;	B	
							NFE: 0.0001440		
*BRCA2* (NM_000059.3)	30	Missense	11/27	c.6101G>A	p.(Arg2034His)	rs80358849	0.000004069;	B	
							NFE: 0.000008973		
*BRCA2* (NM_000059.3)	80	Missense	14/27	c.7021C>T	p.(Arg2341Cys)	rs41293505	0.00002439;	D	
							EA: 0.00005798		
*BRIP1* (NM_032043.2)	68	Missense	15/20	c.2220G>T	p.(Gln740His)	rs45589637	0.0005198;	B	
							L: 0.001395		
*CHEK2* (NM_007194.3	79	Missense	4/15	c.539G>A	p.(Arg180His)	rs137853009	0.00006494;	C	
							L: 0.0002615		
*CHEK2* (NM_007194.3)	83	Missense	6/15	c.688G>C	p.(Ala230Pro)	rs748636216	0.000004063;	D	
							NFE: 0.000008956		
*DDB2* (NM_000107.2)	23	Missense	7/10	c.947C>T	p.(Ser316Phe)	rs375788966	0.00001218;	C	
							SA: 0.00003249		
*DICER1* (NM_030621.4)	8	Missense	25/29	c.4228A>T	p.(Asn1410Tyr)		not found	D	
*DIS3L2* (NM_152383.4)	65	Missense	20/21	c.2450C>T	p.(Thr817Met)	rs376816858	0.00006697;	C	
							SA: 0.0003505		
*EGFR* (NM_005228.3)	54	Missense	17/28	c.2039G>A	p.(Arg680Gln)	rs373336251	0.00009084;	D	
							FE: 0.0003912		
*ERCC2* (NM_000400.3)	24	Disruptive inframe deletion	20/23	c.1857_1859delCAT	p.(Ile619del)		0.000008127;		
							EA: 0.00005800		
*ERCC2* (NM_000400.3)	55	Missense	22/23	c.2083C>T	p.(Arg695Cys)	rs201392911	0.0001372;	D	
							A: 0.0003331		
*ERCC4* (NM_005236.2)	23	Missense	11/11	c.2395C>T	p.(Arg799Trp)	rs121913049	0.0004476;	D	
							NFE: 0.0008138		
*ERCC5* (NM_000123.3)	23	Missense	1/15	c.56C>T	p.(Pro19Leu)	rs34291397	0.0005702;	C	
							NFE: 0.001092		
*ERCC5* (NM_000123.3)	76	Missense	13/15	c.2818G>A	p.(Val940Met)	rs146344855	0.0009378;	C	
							AJ: 0.005122		
*EXT2* (NM_000401.3)	13	Missense	6/14	c.1064G>A	p.(Arg355His)	rs149727518	0.0006422;	D	
							AJ: 0.003448		
*EXT2* (NM_000401.3)	54	Missense	7/14	c.1186G>A	p.(Val396Met)	rs138943091	0.0004148;	D	
							NFE: 0.0007261		
*FANCA* (NM_000135.2)	41, 73	Missense	1/43	c.64T>G	p.(Trp22Gly)		not found	D	
*FANCA* (NM_000135.2)	8	Missense	16/43	c.1489C>G	p.(Pro497Ala)		not found	D	
*FANCA* (NM_000135.2)	27	Missense	22/43	c.2000C > G	p.(Pro667Arg)	rs755293596	0.00002230;	D	
							SA: 0.00004382		
*FANCA* (NM_000135.2)	49	Missense	35/43	c.3430C>T	p.(Arg1144Trp)	rs143671872	0.0005269;	D	
							NFE: 0.0009237		
*FANCA* (NM_000135.2)	54	Missense	37/43	c.3688C>G	p.(Leu1230Val)	rs576401459	0.00002030;	D	
							A: 0.00006535		
*FANCD2* (NM_033084.3)	57	Missense	21/43	c.1933G>T	p.(Asp645Tyr)	rs146496253	0.0001371;	C	
							NFE: 0.0002288		
*FANCI* (NM_001113378.1)	35	Missense	17/38	c.1589T>C	p.(Leu530Pro)	rs766346156	0.000008122;	D	
							SA: 0.00006497		
*GPC3* (NM_001164617.1)	19	Missense	8/9	c.1630C>T	p.(Arg544Cys)	rs759543703	0.00009270;	D	
							A: 0.0001707		
*HNF1A* (NM_000545.6)	48	Missense	5/10	c.1061C>T	p.(Thr354Met)	rs757068809	0.00006495;	D	
							L: 0.0001743		
*KIT* (NM_000222.2)	39	Missense	3/21	c.391G>A	p.(Asp131Asn)		0.00001807;	C	
							A: 0.00004164		
*MET* (NM_001127500.1)	13	Missense	2/21	c.1076G>A	p.(Arg359Gln)	rs201274041	0.0002347;	D	
							NFE: 0.0004298		
*MET* (NM_001127500.1)	75	Missense	14/21	c.3023G>A	p.(Ser1008Asn)		not found	D	
*MLH1* (NM_000249.3)	51	Missense	13/19	c.1457C>T	p.(Ser486Phe)	rs532873141	0.000004061;	C	
							EA: 0.00005798		
*NF1* (NM_001042492)	28	Missense	5/58	c.575G>A	p.(Arg192Gln)	rs587781670	0.00005294;	C	
							EA: 0.0005803		
*NSD1* (NM_022455.4)	10	Missense	23/23	c.7352G>A	p.(Arg2451Lys)	rs200115665	0.0001119;	D	
							NFE: 0.0002212		
*PALB2* (NM_024675.3)	56	Missense	8/13	c.2792T>G	p.(Leu931Arg)	rs773831304	0.00001221;	D	
							NFE: 0.00002694		
*PMS1* (NM_000534.4)	67	Missense	2/13	c.118G>C	p.(Val40Leu)		not found	B	
*PMS1* (NM_000534.4)	12	Missense	3/13	c.287C>A	p.(Ala96Asp)	rs139414606	0.000004063;	D	
							NFE: 0.000008962		
*PTCH1* (NM_001083602.1)	4	Missense	23/24	c.3749A>G	p.(Tyr1250Cys)	rs147067171	0.0005463;	D	
							AJ: 0.001098		
*RAD51C* (NM_058216.2)	42	Splice region	8/8	c.1026+5_1026+7 delGTA		rs747311993	0.00001219;		Native donor site: SSF: − 100.0% MaxEnt: − 100.0% NNSPLICE: − 99.6% HSF: − 14.8%
							NFE: 0.00002687		
*RAD51D* (NM_002878.3)	52	Missense	5/10	c.355T>C	p.(Cys119Arg)	rs201313861	0.00005413;	B	
							L: 0.00008716		
*RHBDF2* (NM_024599.5)	3, 75	Missense	8/19	c.940G>A	p.(Ala314Thr)	rs140433374	0.0007812;	C	
							FE: 0.001173		
*SDHB* (NM_003000.2)	81	Missense	2/8	c.178A>G	p.(Thr60Ala)	rs34599281	0.00006095;	D	
							NFE: 0.0001165		
*SLX4* (NM_032444.2)	14	Missense	14/15	c.4831G>A	p.(Glu1611Lys)	rs766110479	0.00002847;	D	
							EA: 0.00005798		
*TSC1* (NM_000368.4)	51	Missense	12/23	c.1178C>T	p.(Thr393Ile)	rs201452238	0.00002170;	D	
							A: 0.00004165		
*TSC2* (NM_000548.4)	32	Missense	36/42	c.4582G>C	p.(Glu1528Gln)		not found	D	
*WRN* (NM_000553.4)	55	Missense	19/35	c.2131C>T	p.(Arg711Trp)	rs34560788	0.0002057;	D	
							NFE: 0.0003948		

*Abbreviations: HSF* Human Splicing Finder, *SSF* Splice Site Finder

^a^*NFE* European (non-Finnish), *FE* European (Finnish), *A* African, *L* Latino, *EA* East Asian, *SA* South Asian, *AJ* Ashkenazi Jewish, *O* Other

^b^Based on in silico prediction tools Align GVGD, MetaLR, SIFT, and Polymorphism Phenotyping version 2 (PolyPhen-2) (HDIV), as well as phyloP basewise conservation scores; rated as probably damaging (D) with at least 3 of 5 tools predicting damage; rated as probably benign (B) with at least 4 of 5 tools predicting tolerance; or rated as contradictory (C) with 2 of 5 tools predicting damage or conflicting results

Regarding the detection of unconventional, potentially harmful aberrations in *TP53* itself, we did not identify any variants other than commonly known polymorphisms in our cohort. A list of all detected *TP53* variants is accessible in Additional file 4.

## Discussion

In this study, we approached the question whether breast cancer patients with an LFS/LFL-suggestive phenotype may harbor germline aberrations in known or proposed cancer susceptibility genes beyond *TP53* and *BRCA1/2*. Investigating the spectrum of germline mutations in a cohort of 83 BC patients suggestive of LFS/LFL, we identified 10 patients carrying (likely) PVs in the analyzed genes. 3 of these 10 patients carried 2 (likely) PVs each.

### Variants in established BC susceptibility genes (*PALB2, ATM, CHEK2*)

As anticipated beforehand, we detected several PVs in established BC-associated genes. Two women (subjects 7, 76) carry previously described, protein-truncating, heterozygous *PALB2* frameshift variants. The variant PALB2:p.(Gln60Argfs*7), occurring in patient 76, has previously been detected in several unrelated patients with BC [35, 36], whereas the variant PALB2:p.(Arg170Ilefs*14) in patient 7 is a known Polish founder mutation [37]. Notably, patient 76 developed hormone receptor-positive BC disease at the age of 33 years and contralateral BC at age 39 years, whereas patient 7 presented with triple-negative BC, which is in line with studies reporting breast tumors of *PALB2* mutation carriers to be triple-negative in about 34% of these patients [38].

Furthermore, 3 women of our cohort carry heterozygous PVs in *ATM,* one of whom additionally harbors a PV in *CHEK2*. A deletion of the last 2 exons of *ATM* (62–63) was detected in patient 32. Deletions of exon 63 or exons 62–63 of the *ATM* gene have previously been reported in patients with ataxia telangiectasia and are considered to be functionally relevant [39, 40]. In two further patients (60, 40), we detected previously described *ATM* nonsense variants; the variant ATM:p.(Glu1978*) has been reported in BC cases before [41], whereas the variant ATM:p.(Cys2931*) has been described as a class 5 variant in a patient with ataxia telangiectasia [42]. The latter index person (40) additionally harbors a *CHEK2* exon 9–10 deletion, which is a known pathogenic Slavic founder mutation [43]. Remarkably, 2 of the 3 *ATM* mutation carriers of our cohort have first-degree relatives presenting with ovarian cancer, one of whom harbors the additional *CHEK2* exon 9–10 deletion. While monoallelic *ATM* germline PVs have been described in patients with ovarian cancer, heterozygous *ATM* variants have been associated predominantly with elevated BC incidence rather than other cancer types [44, 45].

### Variants in candidate BC susceptibility genes

#### Variants in genes associated with BC in a somatic context (*CDKN2A, RUNX1*)

One woman (patient 58), who is additionally affected by the class 4 splice donor site variant RECQL4:c.1390+1G>C, was identified to carry a *CDKN2A*/p14^ARF^ nonsense variant in exon 2 of 3 total exons [CDKN2A:p.(Arg98*)]. The *CDKN2A* locus encodes two distinct proteins, p14^ARF^ and p16^INK4^, that are defined by translating the common second exon in alternate reading frames. The variant we detected is not predicted to affect expression or function of p16^INK4^, a tumor suppressor implicated in the CDK4/CDK6-RB1 pathway; instead, p14^ARF^ expression is expected to be abolished via nonsense-mediated RNA decay (NMD). The protein p14^ARF^ acts upstream of TP53 by binding directly to MDM2, an E3 ubiquitin ligase controlling the activity and stability of TP53. Because p14^ARF^ promotes MDM2’s degradation, TP53 is stabilized and accumulates, and a TP53 response manifests in elevated levels of p21^CIP1^, inducing cell cycle arrest [46]. PVs in human p14^ARF^ exon 2 have been reported to disrupt its nucleolar localization and impair its ability to block nuclear export of MDM2 and TP53 [47]. The exon 2 variant in our cohort was identified in an index patient whose grandfather was diagnosed with hypopharyngeal carcinoma, which is in line with *CDKN2A* PVs predisposing to tobacco-related cancers such as orolaryngeal cancer, next to its predominant role in hereditary melanoma, pancreatic cancer, and further tumor entities [MIM:600160]. Of note and with particular relevance to LFS and LFL, germline *CDKN2A* PVs have recently been demonstrated to account for a subset (8 of 190) of hereditary sarcoma cases negative for germline *TP53* PVs [17]. Conversely, however, sarcomas are rare in *CDKN2A* mutation carriers, and the affected family of our cohort also does not present with sarcoma cases. In a somatic context, *CDKN2A* PVs have previously been associated with the mutational landscape of BC [48].

In index patient 30, we detected a splice donor site variant in *RUNX1* (RUNX1:c.97+1G>A) predicted deleterious by 4 of 5 splice prediction programs owing to complete loss of the native splice site. As of yet, this variant has not been described in the literature and was classified by us as a class 4 variant on the basis of in silico prediction. *RUNX1* is a gene well known for its crucial role in the hematopoietic system and for its association with sporadic and familial leukemia [49]. The above-mentioned variant was identified in a family exclusively struck by BC. Of note, no hematologic abnormalities were reported in this family at the time of consultation. Even though the variant was detected in 43% of the sequencing reads (VAF 43%, depth 400 ×), we cannot exclude the possibility that this variant might be due to clonal hematopoiesis (i.e., undiagnosed hematologic disorder). In the case of a confirmed germline event, functional splicing assays would be needed to confirm the disruptive nature of the variant. In regard to BC pathology, *RUNX1* has been suggested to be largely understudied [50], and genome-wide sequencing of cohorts of patients with BC have subsequently exposed *RUNX1* as one of the most frequently mutated and/or deleted genes in BC in a somatic setting [51, 52]. Its role in BC progression has been related to estrogen signaling but remains elusive, likely including oncogenic rather than tumor-suppressive functions in mammary epithelial cells [53–55]. A conclusive role for *RUNX1* germline variations in BC pathogenesis cannot be assessed currently and needs further clarification.

#### Variants in Fanconi pathway genes (*FANCI, FANCA*)

The nonsense *FANCI* variant FANCI:p.(Arg1285*) in exon 37 of 38 total exons, located within the last 72 bp of the second to last exon and therefore imprecise in regard to NMD, was detected in patient 65, who was additionally found to carry a deletion of exons 3–8 of the *PMS2* gene, which was verified by MLPA analysis. Because the maternal lineage includes two colorectal carcinomas in two of the index person’s second-degree relatives, diagnosed at 37 and 70 years of age, the *PMS2* variant is primarily suggestive of being responsible for the familial cancer phenotypes. Nonetheless, the *FANCI* variant concerns a highly conserved arginine, has previously been described in patients with Fanconi anemia (FA), and has been found to impair DNA binding and ubiquitination of the ID2 complex, a dimeric complex formed by FANCI and FANCD2, which is relevant for DNA crosslink repair [56, 57]. Because the paternal lineage also shows incidence of malignant disease, it is conceivable that the *FANCI* and *PMS2* variants may jointly combine in the index person’s genome, leading to a more severe phenotype, such as earlier age of onset of disease.

Furthermore, 6 patients of our cohort were identified to carry 5 very rare *FANCA* VUS: FANCA:p.(Trp22Gly) twice, FANCA:p.(Pro497Ala), FANCA:p.(Pro667Arg), FANCA:p.(Arg1144Trp), and FANCA:p.(Leu1230Val). All but one of these rare *FANCA* VUS were absent in a large cohort of 11,000 exome-sequenced individuals of HMGU (Helmholtz Zentrum München – Deutsches Forschungszentrum für Gesundheit und Umwelt) and are either not or very rarely found in datasets from the Genome Aggregation Database [58] (*see* Table 4 for details). Interestingly, clinical presentation of these heterozygous *FANCA* missense VUS carriers comprises some of the most striking LFL features, involving either very early-onset BC or exceptionally Li-Fraumeni-suggestive personal and family traits, such as a woman with triple primaries (malignant hemangiopericytoma at age 12 years, BC at age 28 years, and contralateral BC at age 47 years) or a woman whose sister and half-brother were diagnosed with two liposarcomas and a melanoma, respectively. The median age of onset of BC disease for these 6 *FANC*A VUS carriers was 29.5 years (27, 27, 28, 31, 35, and 44 years), whereas the median age of onset for the entire cohort was 33 years. In a biallelic context, the Fanconi family genes are associated with FA, a condition characterized by congenital abnormalities, bone marrow failure, and cancer predisposition already during childhood. *FANCA* gene PVs are by far the most common in FA, responsible for at least 60% of all cases of FA [59]. Heterozygous parents and siblings of patients with FA have not been found to exhibit an elevated incidence of malignant disease [60]; however, monoallelic *FANCA* variants have been investigated only sparsely in a disease context, and with inconsistent results [61–63]. Considering the rarity of each *FANCA* VUS detected in the present study, and given the possibility of differing *FANCA* missense variants conferring vastly diverse effects on protein function, the disproportionate occurrence of these rare variants appears to be worthy of further investigation.

#### Variants in RECQ helicases (*WRN, RECQL4*)

Two class 4–5 aberrations (patients 59 and 79) and one class 3 copy number change (patient 63) were identified in *WRN, RECQL4,* and *BLM* — 3 of 5 genes belonging to the human family of RecQ helicases, comprising enzymes that drive the unwinding of DNA in an ATP- and Mg^2+^-dependent manner and which are essential for genome maintenance and stability. In an autosomal recessive setting, each of these genes confers very rare and complex syndromes, namely Werner syndrome (*WRN*), Bloom syndrome (*BLM*), and Rothmund-Thomson syndrome or RAPADILINO syndrome (*RECQL4*). In regard to LFS, in which sarcoma incidence is being considered somewhat of a hallmark within the tumor spectrum, a particularly striking fact about the above-mentioned syndromes is that the spectrum of malignancies conferred by them is dominated by sarcomas as well [64, 65]. Moreover, for all three proteins, a firm association with TP53 has been described [65, 66]. Whereas biallelic impairment is considered mandatory for the full spectrum of the syndromes to develop, *WRN* and *BLM* have additionally been suggested as BC susceptibility genes in a monoallelic setting [67–69], and dominant-negative effects or gain-of-function processes have previously been proposed for specific missense mutant WRN or BLM proteins [70].

To our knowledge, this is the first study focusing on a German population-based cohort of familial BC patients suggestive of LFL but negative for causative *TP53* as well as *BRCA1/2* germline PVs to be systematically panel-tested for aberrations in further cancer susceptibility genes. One hypothesis for the emergence of an LFL phenotype without detectable *TP53* PV is the simultaneous and additive occurrence of dual or multiple clearly pathogenic aberrations in more than one cancer susceptibility gene. Our study design was able to confirm double heterozygosity in 3 cases. In regard to patients with BC, double heterozygosity for germline PVs in BC predisposition genes has been detected and discussed before [67]. This type of oligogenic or polygenic model might explain the fact that often the index patients of our cohort happen to be burdened by cancer incidences from both family lineages. The model would also explain why the family mode may not follow a strict autosomal dominant inheritance pattern, which is usually considered a hallmark of classic LFS.

With the current state of knowledge and excluding all VUS, the presently described aberrations, occurring in 10 (12%) of our cohort’s individuals, may in part explain or contribute to some of the corresponding phenotypes. However, the gravity and the spectrum of malignant disease in many of our cohort’s families seem to suggest that additional PVs in modifying genes or as yet unknown cancer predisposition genes may play a role in some of them. Importantly, these results indicate that even after testing a fairly extensive number of cancer susceptibility genes with a panel design covering both sequence alterations and copy number changes, a large number of LFL BC cases remain unexplained. Few studies have approached the quest for further susceptibility genes in individual *TP53*-negative LFS/LFL cases, either by specifically testing single genes associated with the spectrum of tumors appearing in LFS or by performing whole-exome sequencing. Associations have been suggested for LFS-associated brain tumors with nonsense PVs in *CASP9* (caspase-9) [71], for a *POT1* (protection of telomeres 1) missense variant with cardiac and breast angiosarcomas [72], and, as mentioned above, for *CDKN2A* PVs with hereditary sarcoma cases [17].

Apart from the idea of aberrations in additional susceptibility genes being responsible for *TP53*-negative LFL cases, novel mechanisms for *TP53* impairment are also emerging. These include structural variants that cannot easily be detected by conventional approaches such as intron 1 rearrangements [73], variants in the far-off 3′ UTR affecting microRNA binding [74] (a region not covered by the Illumina gene panel used in our study), and novel splicing PVs [75]. Whereas the severe phenotype known for LFS is expected to result predominantly from missense or isoform-specific variants conferring a dominant-negative effect of the mutated over the wild-type proteins, *TP53* haploinsufficiency (e.g., loss of function or gene dosage effects due to splice variants or 3′ UTR variants and deletions, respectively) is emerging as a likely mechanism for a more subtle phenotype, one that may in fact be described as LFL, as has previously been suggested [75]. Testing patients without variants in coding *TP53* regions, such as the ones in our cohort, for the above-mentioned variants would constitute a crucial future undertaking.

The strengths of this study include a well-characterized cohort of LFL BC patients and the dual-method study design of NGS and array-based CGH to cover a larger number of aberrations. Limitations include the following:

1. Segregation analyses would be mandatory in order to characterize the impact of the detected variants in affected and unaffected family members and to predict the penetrance associated with these variants; unfortunately, DNA was not available for any of the family members of affected germline mutation carriers.

2. Breakpoint analysis and, if applicable, translocation details are urgently needed for the *BLM* duplication.

3. Functional assays would be helpful in determining the consequences of the detected VUS and novel aberrations.

## Conclusions

Our study helps define the mutational spectrum in breast cancer patients suggestive of LFS, contributes to a potential relevance of *CDKN2A*/p14^ARF^ in LFS/LFL settings, and points out the need for intensified research on monoallelic variants in Fanconi pathway and RECQ family genes. Notably, our study further reveals that there remains a large portion of unexplained LFS/LFL cases and emphasizes the necessity of advanced research on novel susceptibility genes as well as noncoding *TP53* variants in patients with negative test results for *TP53* variants in coding regions.

## Additional files

Additional file 1: TruSight Cancer Target Genes and SNPs (a list of all 94 investigated genes) (Illumina). (DOCX 15 kb)

Additional file 2: Definitions of Li-Fraumeni criteria (official definitions of available clinical criteria, including classic LFS criteria, LFL criteria of Eeles and Birch, and 3 versions of Chompret criteria). (DOCX 18 kb)

Additional file 3: Pedigrees of investigated families with (likely) pathogenic variants (pedigrees of all families of carriers of pathogenic or likely pathogenic variants in our cohort, including age of onset of malignant disease). (DOCX 692 kb)

Additional file 4: Total list of *TP53* (NM_000546.5) variants detected via NGS-based sequencing (entire list of all detected *TP53* variants in our collective). (DOCX 35 kb)

## Abbreviations

ACMG: American College of Medical Genetics and Genomics
BC: Breast cancer
bp: Base pair(s)
CGH: Comparative genomic hybridization
FA: Fanconi anemia
HBOC: Hereditary breast and ovarian cancer
HMGU: Helmholtz Zentrum München – Deutsches Forschungszentrum für Gesundheit und Umwelt
IGV: Integrative Genomics Viewer
LFL: Li-Fraumeni-like syndrome
LFS: Li-Fraumeni syndrome
MAF: Minor allele frequency
MLPA: Multiplex ligation-dependent probe amplification
NGS: Next-generation sequencing
NMD: Nonsense-mediated RNA decay
PV: Pathogenic variant
ROI: Region of interest
UTR: Untranslated region
VAF: Variant allele frequency
VUS: Variant of unknown significance

## References

[1] Li FP, Fraumeni JF, Mulvihill JJ, Blattner WA, Dreyfus MG, Tucker MA (1988). Miller RW. A cancer family syndrome in twenty-four kindreds. Cancer Res.

[2] Malkin D, Li FP, Strong LC, Fraumeni JF, Nelson CE, Kim DH, Kassel J, Gryka MA, Bischoff FZ, Tainsky MA (1990). Germ line p53 mutations in a familial syndrome of breast cancer, sarcomas, and other neoplasms. Science.

[3] Evans DG, Birch JM, Thorneycroft M, McGown G, Lalloo F, Varley JM (2002). Low rate of TP53 germline mutations in breast cancer/sarcoma families not fulfilling classical criteria for Li-Fraumeni syndrome. J Med Genet.

[4] Birch JM, Hartley AL, Tricker KJ, Prosser J, Condie A, Kelsey AM, Harris M, Jones PH, Binchy A, Crowther D (1994). Prevalence and diversity of constitutional mutations in the p53 gene among 21 Li-Fraumeni families. Cancer Res.

[5] Eeles RA (1995). Germline mutations in the TP53 gene. Cancer Surv.

[6] Chompret A, Abel A, Stoppa-Lyonnet D, Brugieres L, Pages S, Feunteun J, Bonaiti-Pellie C (2001). Sensitivity and predictive value of criteria for p53 germline mutation screening. J Med Genet.

[7] Bougeard G, Sesboue R, Baert-Desurmont S, Vasseur S, Martin C, Tinat J, Brugieres L, Chompret A, de Paillerets BB, Stoppa-Lyonnet D (2008). Molecular basis of the Li-Fraumeni syndrome: an update from the French LFS families. J Med Genet.

[8] Tinat J, Bougeard G, Baert-Desurmont S, Vasseur S, Martin C, Bouvignies E, Caron O, Bressac-de Paillerets B, Berthet P, Dugast C (2009). 2009 Version of the Chompret criteria for Li Fraumeni syndrome. J Clin Oncol.

[9] Bougeard G, Renaux-Petel M, Flaman JM, Charbonnier C, Fermey P, Belotti M, Gauthier-Villars M, Stoppa-Lyonnet D, Consolino E, Brugieres L (2015). Revisiting Li-Fraumeni syndrome from TP53 mutation carriers. J Clin Oncol.

[10] Nichols KE, Malkin D, Garber JE, Fraumeni JF, Li FP (2001). Germ-line p53 mutations predispose to a wide spectrum of early-onset cancers. Cancer Epidemiol Biomark Prev.

[11] Chompret A, Brugieres L, Ronsin M, Gardes M, Dessarps-Freichey F, Abel A, Hua D, Ligot L, Dondon MG, Bressac-de Paillerets B (2000). P53 germline mutations in childhood cancers and cancer risk for carrier individuals. Br J Cancer.

[12] Wooster R, Weber BL (2003). Breast and ovarian cancer. N Engl J Med.

[13] Gonzalez KD, Noltner KA, Buzin CH, Gu D, Wen-Fong CY, Nguyen VQ, Han JH, Lowstuter K, Longmate J, Sommer SS (2009). Beyond Li Fraumeni syndrome: clinical characteristics of families with p53 germline mutations. J Clin Oncol.

[14] McCuaig JM, Armel SR, Novokmet A, Ginsburg OM, Demsky R, Narod SA, Malkin D (2012). Routine TP53 testing for breast cancer under age 30: ready for prime time?. Fam Cancer.

[15] Kraus C, Hoyer J, Vasileiou G, Wunderle M, Lux MP, Fasching PA, Krumbiegel M, Uebe S, Reuter M, Beckmann MW (2017). Gene panel sequencing in familial breast/ovarian cancer patients identifies multiple novel mutations also in genes others than BRCA1/2. Int J Cancer.

[16] Slavin TP, Maxwell KN, Lilyquist J, Vijai J, Neuhausen SL, Hart SN, Ravichandran V, Thomas T, Maria A, Villano D (2017). The contribution of pathogenic variants in breast cancer susceptibility genes to familial breast cancer risk. NPJ Breast Cancer.

[17] Jouenne F, Chauvot de Beauchene I, Bollaert E, Avril MF, Caron O, Ingster O, Lecesne A, Benusiglio P, Terrier P, Caumette V (2017). Germline *CDKN2A*/P16^INK4A^ mutations contribute to genetic determinism of sarcoma. J Med Genet.

[18] Meindl A, Ditsch N, Kast K, Rhiem K, Schmutzler RK (2011). Hereditary breast and ovarian cancer: new genes, new treatments, new concepts. Dtsch Arztebl Int.

[19] megSAP – Medical Genetics Sequence Analysis Pipeline. https://github.com/imgag/megSAP.

[20] Prochazkova K, Pavlikova K, Minarik M, Sumerauer D, Kodet R, Sedlacek Z (2009). Somatic TP53 mutation mosaicism in a patient with Li-Fraumeni syndrome. Am J Med Genet A.

[21] Robinson JT, Thorvaldsdottir H, Winckler W, Guttman M, Lander ES, Getz G, Mesirov JP (2011). Integrative genomics viewer. Nat Biotechnol.

[22] Thorvaldsdottir H, Robinson JT, Mesirov JP (2013). Integrative Genomics Viewer (IGV): high-performance genomics data visualization and exploration. Brief Bioinform.

[23] ngs-bits – Short-read sequencing tools: GSvar. https://github.com/imgag/ngs-bits.

[24] Auton A, Brooks LD, Durbin RM, Garrison EP, Kang HM, Korbel JO, Marchini JL, McCarthy S, McVean GA (2015). Abecasis GR. A global reference for human genetic variation. Nature.

[25] Lek M, Karczewski KJ, Minikel EV, Samocha KE, Banks E, Fennell T, O’Donnell-Luria AH, Ware JS, Hill AJ, Cummings BB (2016). Analysis of protein-coding genetic variation in 60,706 humans. Nature.

[26] Glusman G, Caballero J, Mauldin DE, Hood L, Roach JC (2011). Kaviar: an accessible system for testing SNV novelty. Bioinformatics.

[27] Dong C, Wei P, Jian X, Gibbs R, Boerwinkle E, Wang K, Liu X (2015). Comparison and integration of deleteriousness prediction methods for nonsynonymous SNVs in whole exome sequencing studies. Hum Mol Genet.

[28] Kumar P, Henikoff S, Ng PC (2009). Predicting the effects of coding non-synonymous variants on protein function using the SIFT algorithm. Nat Protoc.

[29] Adzhubei IA, Schmidt S, Peshkin L, Ramensky VE, Gerasimova A, Bork P, Kondrashov AS, Sunyaev SR (2010). A method and server for predicting damaging missense mutations. Nat Methods.

[30] Landrum MJ, Lee JM, Benson M, Brown G, Chao C, Chitipiralla S, Gu B, Hart J, Hoffman D, Hoover J (2016). ClinVar: public archive of interpretations of clinically relevant variants. Nucleic Acids Res.

[31] Stenson PD, Ball EV, Mort M, Phillips AD, Shiel JA, Thomas NS, Abeysinghe S, Krawczak M, Cooper DN (2003). Human Gene Mutation Database (HGMD): 2003 update. Hum Mutat.

[32] FLOSSIES: a database of germline genomic variation in healthy older women. https://whi.color.com/.

[33] Richards S, Aziz N, Bale S, Bick D, Das S, Gastier-Foster J, Grody WW, Hegde M, Lyon E, Spector E (2015). Standards and guidelines for the interpretation of sequence variants: a joint consensus recommendation of the American College of Medical Genetics and Genomics and the Association for Molecular Pathology. Genet Med.

[34] Hackmann K, Kuhlee F, Betcheva-Krajcir E, Kahlert AK, Mackenroth L, Klink B, Di Donato N, Tzschach A, Kast K, Wimberger P (2016). Ready to clone: CNV detection and breakpoint fine-mapping in breast and ovarian cancer susceptibility genes by high-resolution array CGH. Breast Cancer Res Treat.

[35] Janatova M, Kleibl Z, Stribrna J, Panczak A, Vesela K, Zimovjanova M, Kleiblova P, Dundr P, Soukupova J, Pohlreich P (2013). The *PALB2* gene is a strong candidate for clinical testing in *BRCA1*- and *BRCA2*-negative hereditary breast cancer. Cancer Epidemiol Biomark Prev.

[36] Thompson ER, Gorringe KL, Rowley SM, Wong-Brown MW, McInerny S, Li N, Trainer AH, Devereux L, Doyle MA, Li J (2015). Prevalence of *PALB2* mutations in Australian familial breast cancer cases and controls. Breast Cancer Res.

[37] Dansonka-Mieszkowska A, Kluska A, Moes J, Dabrowska M, Nowakowska D, Niwinska A, Derlatka P, Cendrowski K, Kupryjanczyk J (2010). A novel germline *PALB2* deletion in Polish breast and ovarian cancer patients. BMC Med Genet.

[38] Cybulski C, Kluzniak W, Huzarski T, Wokolorczyk D, Kashyap A, Jakubowska A, Szwiec M, Byrski T, Debniak T, Gorski B (2015). Clinical outcomes in women with breast cancer and a *PALB2* mutation: a prospective cohort analysis. Lancet Oncol.

[39] Huang Y, Yang L, Wang J, Yang F, Xiao Y, Xia R, Yuan X, Yan M (2013). Twelve novel *Atm* mutations identified in Chinese ataxia telangiectasia patients. Neuromolecular Med.

[40] Podralska MJ, Stembalska A, Slezak R, Lewandowicz-Uszynska A, Pietrucha B, Koltan S, Wigowska-Sowinska J, Pilch J, Mosor M, Ziolkowska-Suchanek I (2014). Ten new *ATM* alterations in Polish patients with ataxia-telangiectasia. Mol Genet Genomic Med.

[41] Bogdanova N, Cybulski C, Bermisheva M, Datsyuk I, Yamini P, Hillemanns P, Antonenkova NN, Khusnutdinova E, Lubinski J, Dork T (2009). A nonsense mutation (E1978X) in the *ATM* gene is associated with breast cancer. Breast Cancer Res Treat.

[42] Sandoval N, Platzer M, Rosenthal A, Dork T, Bendix R, Skawran B, Stuhrmann M, Wegner RD, Sperling K, Banin S (1999). Characterization of *ATM* gene mutations in 66 ataxia telangiectasia families. Hum Mol Genet.

[43] Cybulski C, Wokolorczyk D, Huzarski T, Byrski T, Gronwald J, Gorski B, Debniak T, Masojc B, Jakubowska A, Gliniewicz B (2006). A large germline deletion in the Chek2 kinase gene is associated with an increased risk of prostate cancer. J Med Genet.

[44] Minion LE, Dolinsky JS, Chase DM, Dunlop CL, Chao EC, Monk BJ (2015). Hereditary predisposition to ovarian cancer, looking beyond BRCA1/BRCA2. Gynecol Oncol.

[45] Thompson D, Duedal S, Kirner J, McGuffog L, Last J, Reiman A, Byrd P, Taylor M, Easton DF (2005). Cancer risks and mortality in heterozygous *ATM* mutation carriers. J Natl Cancer Inst.

[46] Zhang Y, Xiong Y, Yarbrough WG (1998). ARF promotes MDM2 degradation and stabilizes p53: *ARF-INK4a* locus deletion impairs both the Rb and p53 tumor suppression pathways. Cell.

[47] Zhang Y, Xiong Y (1999). Mutations in human ARF exon 2 disrupt its nucleolar localization and impair its ability to block nuclear export of MDM2 and p53. Mol Cell.

[48] Pereira B, Chin SF, Rueda OM, Vollan HK, Provenzano E, Bardwell HA, Pugh M, Jones L, Russell R, Sammut SJ (2016). The somatic mutation profiles of 2,433 breast cancers refines their genomic and transcriptomic landscapes. Nat Commun.

[49] Schlegelberger B, Heller PG (2017). RUNX1 deficiency (familial platelet disorder with predisposition to myeloid leukemia, FPDMM). Semin Hematol.

[50] Janes KA (2011). RUNX1 and its understudied role in breast cancer. Cell Cycle.

[51] Ellis MJ, Ding L, Shen D, Luo J, Suman VJ, Wallis JW, Van Tine BA, Hoog J, Goiffon RJ, Goldstein TC (2012). Whole-genome analysis informs breast cancer response to aromatase inhibition. Nature.

[52] Banerji S, Cibulskis K, Rangel-Escareno C, Brown KK, Carter SL, Frederick AM, Lawrence MS, Sivachenko AY, Sougnez C, Zou L (2012). Sequence analysis of mutations and translocations across breast cancer subtypes. Nature.

[53] Browne G, Taipaleenmaki H, Bishop NM, Madasu SC, Shaw LM, van Wijnen AJ, Stein JL, Stein GS, Lian JB (2015). Runx1 is associated with breast cancer progression in MMTV-PyMT transgenic mice and its depletion in vitro inhibits migration and invasion. J Cell Physiol.

[54] Chimge NO, Frenkel B (2013). The RUNX family in breast cancer: relationships with estrogen signaling. Oncogene.

[55] Ferrari N, Mohammed ZM, Nixon C, Mason SM, Mallon E, McMillan DC, Morris JS, Cameron ER, Edwards J, Blyth K (2014). Expression of RUNX1 correlates with poor patient prognosis in triple negative breast cancer. PLoS One.

[56] Smogorzewska A, Matsuoka S, Vinciguerra P, McDonald ER, Hurov KE, Luo J, Ballif BA, Gygi SP, Hofmann K, D’Andrea AD (2007). Identification of the FANCI protein, a monoubiquitinated FANCD2 paralog required for DNA repair. Cell.

[57] Longerich S, Kwon Y, Tsai MS, Hlaing AS, Kupfer GM, Sung P (2014). Regulation of FANCD2 and FANCI monoubiquitination by their interaction and by DNA. Nucleic Acids Res.

[58] gnomAD – genome Aggregation Database. http://gnomad.broadinstitute.org/.

[59] Shimamura A, Alter BP (2010). Pathophysiology and management of inherited bone marrow failure syndromes. Blood Rev.

[60] Tischkowitz M, Easton DF, Ball J, Hodgson SV, Mathew CG (2008). Cancer incidence in relatives of British Fanconi anaemia patients. BMC Cancer.

[61] Solyom S, Winqvist R, Nikkila J, Rapakko K, Hirvikoski P, Kokkonen H, Pylkas K (2011). Screening for large genomic rearrangements in the FANCA gene reveals extensive deletion in a Finnish breast cancer family. Cancer Lett.

[62] Litim N, Labrie Y, Desjardins S, Ouellette G, Plourde K, Belleau P, Durocher F (2013). Polymorphic variations in the FANCA gene in high-risk non-BRCA1/2 breast cancer individuals from the French Canadian population. Mol Oncol.

[63] Abbasi S, Rasouli M (2017). A rare FANCA gene variation as a breast cancer susceptibility allele in an Iranian population. Mol Med Rep.

[64] Calvert GT, Randall RL, Jones KB, Cannon-Albright L, Lessnick S, Schiffman JD (2012). At-risk populations for osteosarcoma: the syndromes and beyond. Sarcoma.

[65] Nakayama H (2002). RecQ family helicases: roles as tumor suppressor proteins. Oncogene.

[66] De S, Kumari J, Mudgal R, Modi P, Gupta S, Futami K, Goto H, Lindor NM, Furuichi Y, Mohanty D (2012). RECQL4 is essential for the transport of p53 to mitochondria in normal human cells in the absence of exogenous stress. J Cell Sci.

[67] Sokolenko AP, Bogdanova N, Kluzniak W, Preobrazhenskaya EV, Kuligina ES, Iyevleva AG, Aleksakhina SN, Mitiushkina NV, Gorodnova TV, Bessonov AA (2014). Double heterozygotes among breast cancer patients analyzed for BRCA1, CHEK2, ATM, NBN/NBS1, and BLM germ-line mutations. Breast Cancer Res Treat.

[68] Prokofyeva D, Bogdanova N, Dubrowinskaja N, Bermisheva M, Takhirova Z, Antonenkova N, Turmanov N, Datsyuk I, Gantsev S, Christiansen H (2013). Nonsense mutation p.Q548X in BLM, the gene mutated in Bloom’s syndrome, is associated with breast cancer in Slavic populations. Breast Cancer Res Treat.

[69] Wang Z, Xu Y, Tang J, Ma H, Qin J, Lu C, Wang X, Hu Z, Shen H (2009). A polymorphism in Werner syndrome gene is associated with breast cancer susceptibility in Chinese women. Breast Cancer Res Treat.

[70] Wu Y, Brosh RM (2010). Helicase-inactivating mutations as a basis for dominant negative phenotypes. Cell Cycle.

[71] Ronellenfitsch MW, Oh JE, Satomi K, Sumi K, Harter PN, Steinbach JP, Felsberg J, Capper D, Voegele C, Durand G (2018). *CASP9* germline mutation in a family with multiple brain tumors. Brain Pathol.

[72] Calvete O, Martinez P, Garcia-Pavia P, Benitez-Buelga C, Paumard-Hernandez B, Fernandez V, Dominguez F, Salas C, Romero-Laorden N, Garcia-Donas J (2015). A mutation in the *POT1* gene is responsible for cardiac angiosarcoma in TP53-negative Li-Fraumeni-like families. Nat Commun.

[73] Ribi S, Baumhoer D, Lee K, Edison, Teo AS, Madan B, Zhang K, Kohlmann WK, Yao F, Lee WH (2015). TP53 intron 1 hotspot rearrangements are specific to sporadic osteosarcoma and can cause Li-Fraumeni syndrome. Oncotarget.

[74] Macedo GS, Araujo Vieira I, Brandalize AP, Giacomazzi J, Inez Palmero E, Volc S, Rodrigues Paixao-Cortes V, Caleffi M, Silva Alves M, Achatz MI (2016). Rare germline variant (rs78378222) in the TP53 3′ UTR: evidence for a new mechanism of cancer predisposition in Li-Fraumeni syndrome. Cancer Genet.

[75] Piao J, Sakurai N, Iwamoto S, Nishioka J, Nakatani K, Komada Y, Mizutani S, Takagi M (2013). Functional studies of a novel germline p53 splicing mutation identified in a patient with Li-Fraumeni-like syndrome. Mol Carcinog.

