# Supplementary material for: Breast cancer patients suggestive of Li-Fraumeni syndrome: mutational spectrum, candidate genes, and unexplained heredity
Source: Breast Cancer Res. 2018 Aug 7;20:87. doi: 10.1186/s13058-018-1011-1 (PMC6081832; doi:10.1186/s13058-018-1011-1)
Supplement: Supplementary file 1 — TruSight Cancer Target Genes and SNPs (a list of all 94 investigated genes) (Illumina). (DOCX 15 kb) [file 13058_2018_1011_MOESM1_ESM.docx]

**Additional file 1**

**TruSight Cancer Target Genes and SNPs**

Illumina, San Diego, CA, USA

**Genes**

*AIP, ALK, APC, ATM, BAP1, BLM, BMPR1A, BRCA1, BRCA2, BRIP1, BUB1B, CDC73, CDH1, CDK4, CDKN1C, CDKN2A, CEBPA, CEP57, CHEK2, CYLD, DDB2, DICER1, DIS3L2, EGFR, EPCAM, ERCC2, ERCC3, ERCC4, ERCC5, EXT1, EXT2, EZH2, FANCA, FANCB, FANCC, FANCD2, FANCE, FANCF, FANCG, FANCI, FANCL, FANCM, FH, FLCN, GATA2, GPC3, HNF1A, HRAS, KIT, MAX, MEN1, MET, MLH1, MSH2, MSH6, MUTYH, NBN, NF1, NF2, NSD1, PALB2, PHOX2B, PMS1, PMS2, PRF1, PRKAR1A, PTCH1, PTEN, RAD51C, RAD51D, RB1, RECQL4, RET, RHBDF2, RUNX1, SBDS, SDHAF2, SDHB, SDHC, SDHD, SLX4, SMAD4, SMARCB1, STK11, SUFU, TMEM127, TP53, TSC1, TSC2, VHL, WRN, WT1, XPA,* and *XPC*

**reference SNPs**

rs17401966, rs9430161, rs7538876, rs11249433, rs7412746, rs3790844, rs6691170, rs6687758, rs801114, rs1465618, rs7579899, rs1432295, rs721048, rs10187424, rs17483466, rs12621278, rs2072590, rs13016963, rs13393577, rs3768716, rs6435862, rs13387042, rs966423, rs13397985, rs7584330, rs2292884, rs757978, rs4973768, rs1052501, rs2660753, rs9284813, rs17181170, rs9841504, rs10934853, rs6763931, rs6774494, rs10936599, rs10936632, rs4488809, rs10937405, rs17505102, rs710521, rs2131877, rs798766, rs1494961, rs12500426, rs17021918, rs1229984, rs971074, rs7679673, rs10069690, rs2242652, rs2736100, rs2853676, rs4635969, rs4975616, rs401681, rs31489, rs12653946, rs2255280, rs13361707, rs2121875, rs4415084, rs889312, rs10052657, rs20541, rs4624820, rs10058728, rs872071, rs12210050, rs4712653, rs6939340, rs4324798, rs29232, rs3129055, rs2860580, rs2517713, rs6457327, rs130067, rs2894207, rs2596542, rs2248462, rs3117582, rs204999, rs9268542, rs6903608, rs2395185, rs2858870, rs674313, rs28421666, rs2647012, rs10484561, rs9275572, rs210138, rs10484761, rs339331, rs2180341, rs9485372, rs2046210, rs651164, rs9364554, rs7758229, rs4487645, rs11978267, rs4132601, rs6465657, rs1495741, rs1512268, rs2439302, rs16892766, rs1016343, rs1456315, rs16901979, rs2456449, rs16902094, rs445114, rs13281615, rs1562430, rs10505477, rs6983267, rs7014346, rs1447295, rs4242382, rs4242384, rs7837688, rs9642880, rs2019960, rs10088218, rs891835, rs4295627, rs2294008, rs7040024, rs755383, rs3814113, rs7023329, rs2157719, rs1412829, rs1011970, rs4977756, rs965513, rs865686, rs505922, rs10795668, rs11012732, rs3123078, rs10993994, rs10821936, rs7089424, rs10822013, rs10995190, rs224278, rs704010, rs3765524, rs2274223, rs3781264, rs17119461, rs12413624, rs11199874, rs2981579, rs2981575, rs1219648, rs2981582, rs3817198, rs7127900, rs110419, rs1945213, rs11228565, rs7931342, rs10896449, rs7130881, rs7105934, rs614367, rs1393350, rs1801516, rs3802842, rs498872, rs735665, rs2900333, rs718314, rs10875943, rs11169552, rs902774, rs995030, rs3782181, rs4474514, rs11066015, rs671, rs4767364, rs2074356, rs11066280, rs4765623, rs1572072, rs9510787, rs753955, rs9600079, rs9573163, rs9543325, rs7335046, rs944289, rs116909374, rs4444235, rs4779584, rs4924410, rs4775302, rs8030672, rs7176508, rs8034191, rs1051730, rs8042374, rs3803662, rs4784227, rs3112612, rs9929218, rs391525, rs258322, rs1805007, rs4785763, rs4795519, rs4430796, rs7501939, rs7210100, rs1859962, rs17674580, rs7238033, rs4939827, rs8170, rs8102137, rs10411210, rs8102476, rs11083846, rs2735839, rs961253, rs910873, rs4925386, rs6010620, rs4809324, rs372883, rs2014300, rs45430, rs1547374, rs738722, rs36600, rs2284063, rs1014971, rs5759167, rs5768709, rs1327301, rs5945572, rs5945619, rs5919432, rs1321311, rs3824999, rs5934683, rs2283873, rs807624, rs1027643, rs3755132, rs790356, rs5955543, rs10974944, rs1210110, rs7555566, rs1364054, rs6734275, rs7584993, rs17272796, rs1155741, rs161792, rs11940551, rs9293511, rs9352613, rs685449, rs7808249, rs1106334, rs11017876, rs9572094, rs4905366, rs4775699, rs1528601, rs11655512, rs4793172, rs242076, rs6603251, AMG_mid100, MITF_rs149617956, ATM_SNP, and HOXB13_rs138213197
