# Supplementary material for: Breast cancer patients suggestive of Li-Fraumeni syndrome: mutational spectrum, candidate genes, and unexplained heredity
Source: Breast Cancer Res. 2018 Aug 7;20:87. doi: 10.1186/s13058-018-1011-1 (PMC6081832; doi:10.1186/s13058-018-1011-1)
Supplement: Supplementary file 2 — Definitions of Li-Fraumeni criteria (official definitions of available clinical criteria, including classic LFS criteria, LFL criteria of Eeles and Birch, and 3 versions of Chompret criteria). (DOCX 18 kb) [file 13058_2018_1011_MOESM2_ESM.docx]

**Additional file 2**

**Definitions of Li-Fraumeni criteria**

Classic Li-Fraumeni syndrome (LFS) criteria [s1]

A proband with a sarcoma diagnosed before the age of 45 years AND

A first-degree relative with any cancer diagnosed before the age of 45 years AND

Another first- or second-degree relative with any cancer before age 45 or a sarcoma at any age

Li-Fraumeni-like syndrome (LFL) criteria (Birch) [s2]

A proband with any childhood cancer or sarcoma, brain tumor, or adrenocortical carcinoma diagnosed before the age of 45 years AND

A first- or second-degree relative with a typical LFS-related cancer (sarcoma, breast cancer, brain tumor, leukemia, or adrenocortical carcinoma) diagnosed at any age AND

A first- or second-degree relative in the same lineage with any cancer diagnosed before the age of 60 years

Li-Fraumeni-like syndrome **(**LFL) criteria (Eeles) [s3]

Two different tumors that are part of extended LFS (sarcoma, breast cancer, brain tumor, leukemia, adrenocortical carcinoma, melanoma, prostate cancer, and pancreatic cancer) in first- or second-degree relatives at any age

Original Chompret criteria [s4]

A proband affected by a narrow spectrum cancer (sarcoma, brain tumor, breast cancer, and adrenocortical carcinoma) before 36 years AND

At least one first- or second-degree relative affected by a narrow spectrum tumor (other than breast cancer if the proband is affected by breast cancer) before 46 years or with multiple primary tumors at any age

OR

A proband with multiple primary tumors, two of which belong to the narrow spectrum cancers (see above), with the initial cancer occurring before 36 years, regardless of family history

OR

A proband with adrenocortical carcinoma, regardless of age of onset and family history

Proposed Revised Chompret criteria (2008 version) [s5]

A proband with a tumor belonging to the LFS tumor spectrum (soft tissue sarcoma, osteosarcoma, brain tumor, pre-menopausal breast cancer, adrenocortical carcinoma, leukaemia, lung bronchoalveolar cancer) before 46 years AND at least one first or second degree relative with an LFS tumor (except breast cancer if the proband is affected by breast cancer) before 56 years or with multiple tumors

OR

A proband with multiple tumors, two of which belong to the narrow LFS tumor spectrum and the first of which occurred before 46 years

OR

A patient with adrenocortical carcinoma or a patient with breast cancer before 36 years of age without BRCA mutation, irrespective of the family history

Revised Chompret criteria (2009 version) [s6]

A proband with a tumor belonging to LFS tumor spectrum (sarcoma, brain tumor, breast cancer, adrenocortical carcinoma, leukemia, lung bronchoalveolar cancer) before age 46 years AND at least one first or second degree relative with LFS tumor (except breast cancer if the proband is affected by breast cancer) before age 56 years or with multiple primary tumors

OR

A proband with multiple primary tumors (except multiple breast tumors), two of which belong to LFS tumor spectrum and the first of which occurred before age 46 years

OR

A patient with adrenocortical carcinoma or choroid plexus carcinoma, irrespective of family history

Updated Chompret criteria (2015 version) [s7]

A proband with a tumor belonging to LFS tumor spectrum (e.g. premenopausal breast cancer, soft tissue sarcoma, osteosarcoma, CNS tumor, adrenocortical carcinoma) before age 46 years, AND at least one first or second-degree relative with LFS tumor (except breast cancer if proband is affected by breast cancer) before age 56 years or with multiple tumors

OR

Proband with multiple tumors (except multiple breast tumors), two of which belong to LFS tumor spectrum and first of which occurred before age 46 years

OR

Patient with adrenocortical carcinoma, choroid plexus tumor, or rhabdomyosarcoma of embryonal anaplastic subtype, irrespective of family history

OR

Breast cancer before age 31 years

s1. Li FP, Fraumeni JF, Jr., Mulvihill JJ, Blattner WA, Dreyfus MG, Tucker MA and Miller RW. A cancer family syndrome in twenty-four kindreds. Cancer research. 1988; 48(18):5358-5362.

s2. Birch JM, Hartley AL, Tricker KJ, Prosser J, Condie A, Kelsey AM, Harris M, Jones PH, Binchy A, Crowther D and et al. Prevalence and diversity of constitutional mutations in the p53 gene among 21 Li-Fraumeni families. Cancer research. 1994; 54(5):1298-1304.

s3. Eeles RA. Germline mutations in the TP53 gene. Cancer surveys. 1995; 25:101-124.

s4. Chompret A, Abel A, Stoppa-Lyonnet D, Brugieres L, Pages S, Feunteun J and Bonaiti-Pellie C. Sensitivity and predictive value of criteria for p53 germline mutation screening. Journal of medical genetics. 2001; 38(1):43-47.

S5. Bougeard G, Sesboue R, Baert-Desurmont S, Vasseur S, Martin C, Tinat J, Brugieres L, Chompret A, de Paillerets BB, Stoppa-Lyonnet D et al. Molecular basis of the Li-Fraumeni syndrome: an update from the French LFS families. J Med Genet. 2008;45(8):535-8.

S6. Tinat J, Bougeard G, Baert-Desurmont S, Vasseur S, Martin C, Bouvignies E, Caron O, Bressac-de Paillerets B, Berthet P, Dugast C, Bonaiti-Pellie C, Stoppa-Lyonnet D and Frebourg T. 2009 version of the Chompret criteria for Li Fraumeni syndrome. Journal of clinical oncology : official journal of the American Society of Clinical Oncology. 2009; 27(26):e108-109; author reply e110.

S7. Bougeard G, Renaux-Petel M, Flaman JM, Charbonnier C, Fermey P, Belotti M, Gauthier-Villars M, Stoppa-Lyonnet D, Consolino E, Brugieres L, Caron O, Benusiglio PR, Bressac-de Paillerets B, Bonadona V, Bonaiti-Pellie C, Tinat J, et al. Revisiting Li-Fraumeni Syndrome From TP53 Mutation Carriers. Journal of clinical oncology : official journal of the American Society of Clinical Oncology. 2015; 33(21):2345-2352.
