# Supplementary material for: Breast cancer patients suggestive of Li-Fraumeni syndrome: mutational spectrum, candidate genes, and unexplained heredity
Source: Breast Cancer Res. 2018 Aug 7;20:87. doi: 10.1186/s13058-018-1011-1 (PMC6081832; doi:10.1186/s13058-018-1011-1)
Supplement: Supplementary file 3 — Pedigrees of investigated families with (likely) pathogenic variants (pedigrees of all families of carriers of pathogenic or likely pathogenic variants in our cohort, including age of onset of malignant disease). (DOCX 692 kb) [file 13058_2018_1011_MOESM3_ESM.docx]

**Additional file 3**

**Pedigrees of investigated families with (likely) pathogenic variants**

The index patient is marked by an arrow. Index patient ID and gene variants identified in the index patient are specified below the pedigree. BC, breast cancer (dark-filled symbol); OvCa, ovarian cancer (dark-filled circle within the symbol); LFS/LFL-associated tumors (dark-filled lower right corner within the symbol); bilat., bilateral.

1. Patient #7, PALB2:p.(Arg170Ilefs*14)
2. Patient #43, RUNX1:c.97+1G>A
3. Patient #45, *ATM* exon 62-63 del
4. Patient #55, ATM:p.(Cys2931*), *CHEK2* exon 9-10 del
5. Patient #77, CDKN2A:p.(Arg98*), RECQL4:c.1390+1G>C
6. Patient #78, *WRN* exon 15-16 del
7. Patient #79, ATM:p.(Glu1978*)
8. Patient #84, *PMS2* exon 3-8 del, FANCI:p.(Arg1285*)
9. Patient #96, PALB2:p.(Gln60Argfs*7)
10. Patient #99, RECQL4:p.(Ala919Thr)


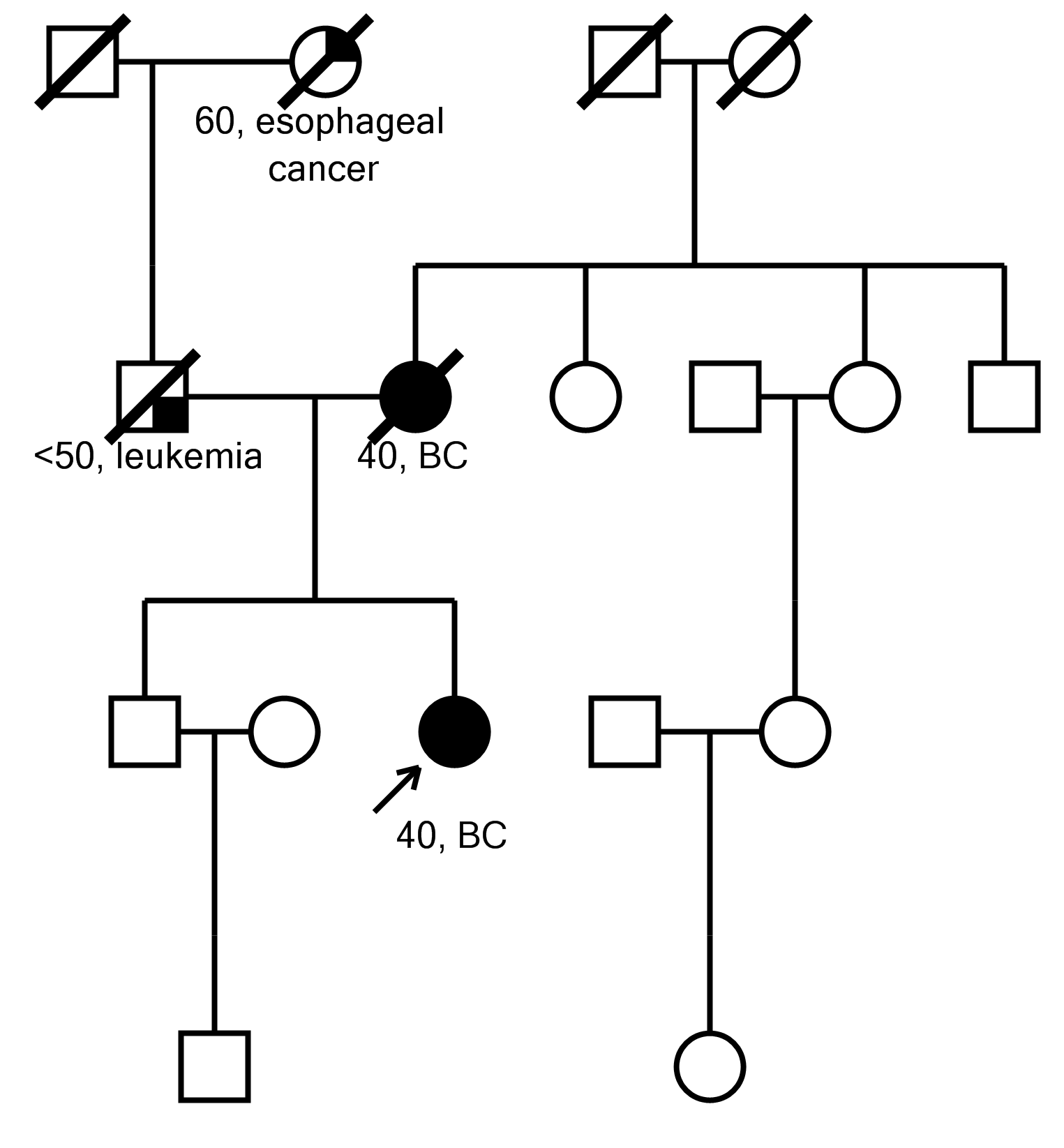


1. Patient #7, PALB2:p.(Arg170Ilefs*14)


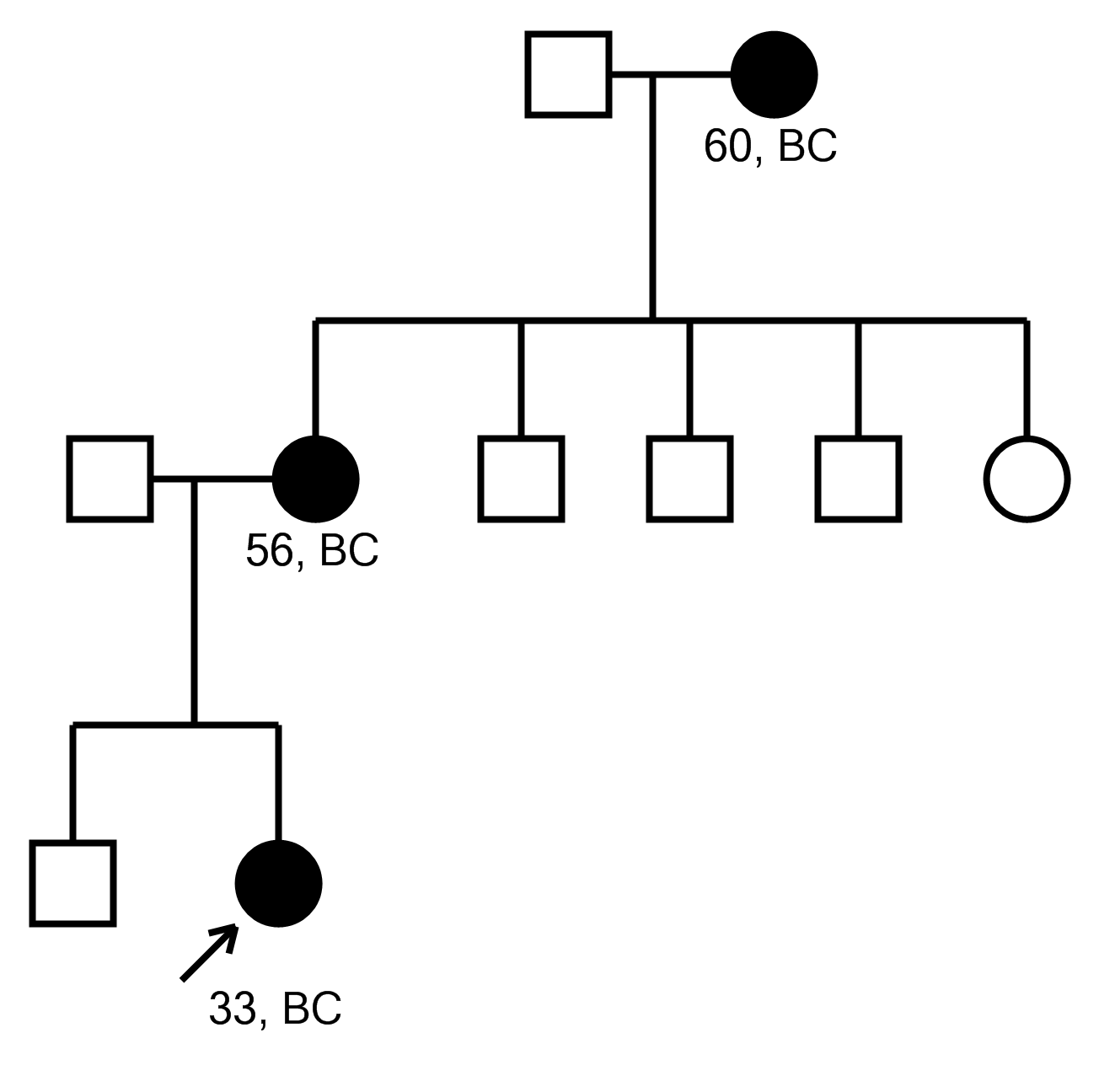


1. Patient #43, RUNX1:c.97+1G>A


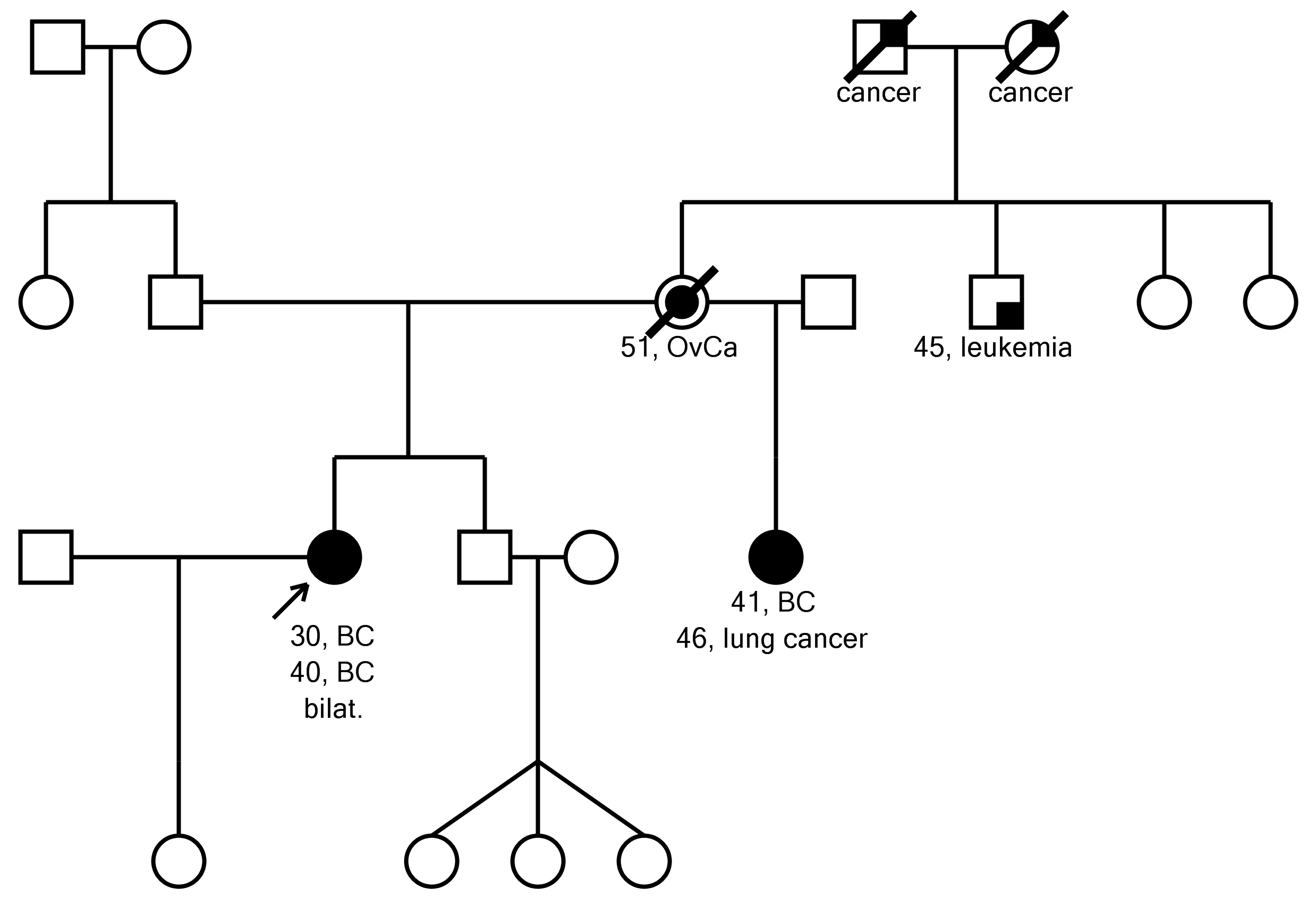


1. Patient #45, *ATM* exon 62-63 del


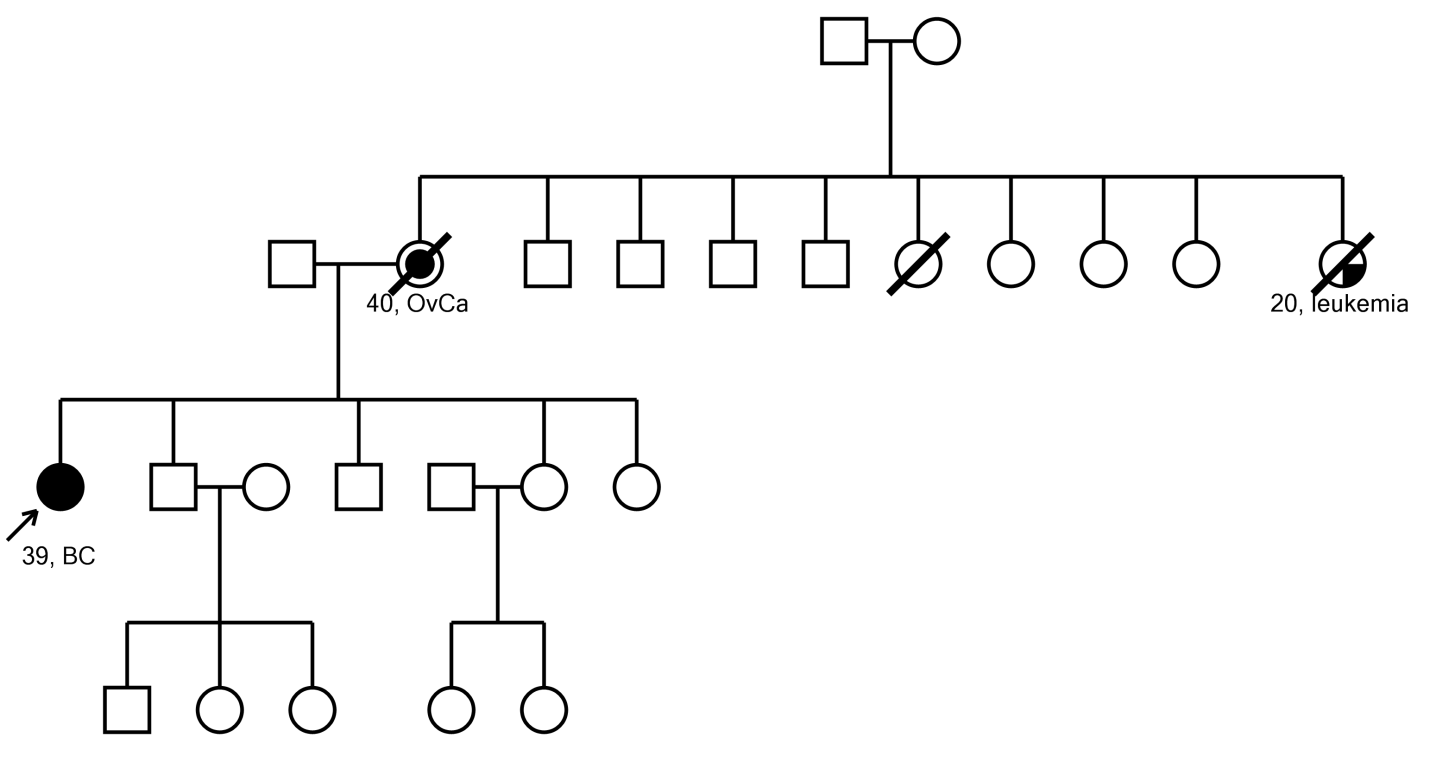


1. Patient #55, ATM:p.(Cys2931*), *CHEK2* exon 9-10 del


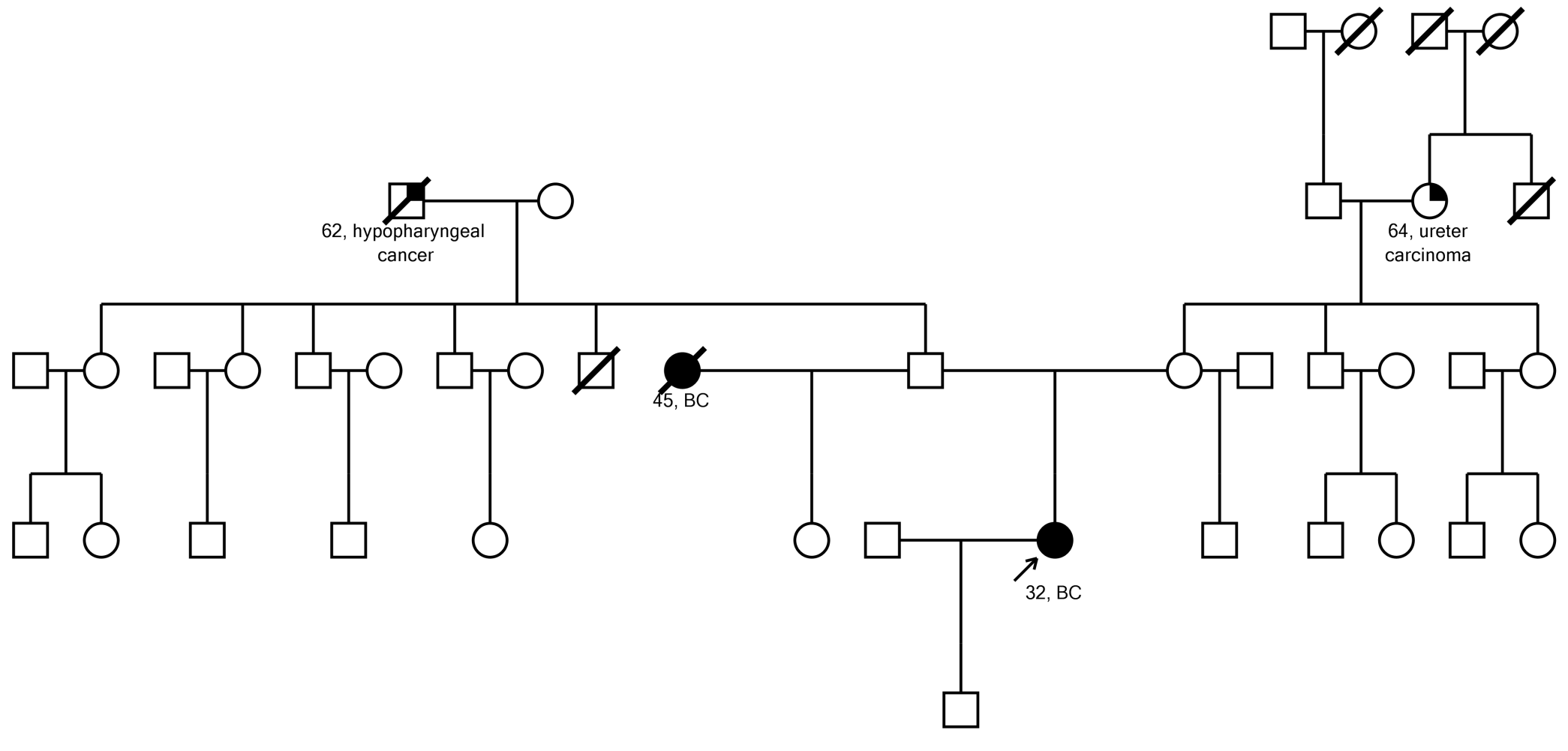


1. Patient #77, CDKN2A:p.(Arg98*), RECQL4:c.1390+1G>C


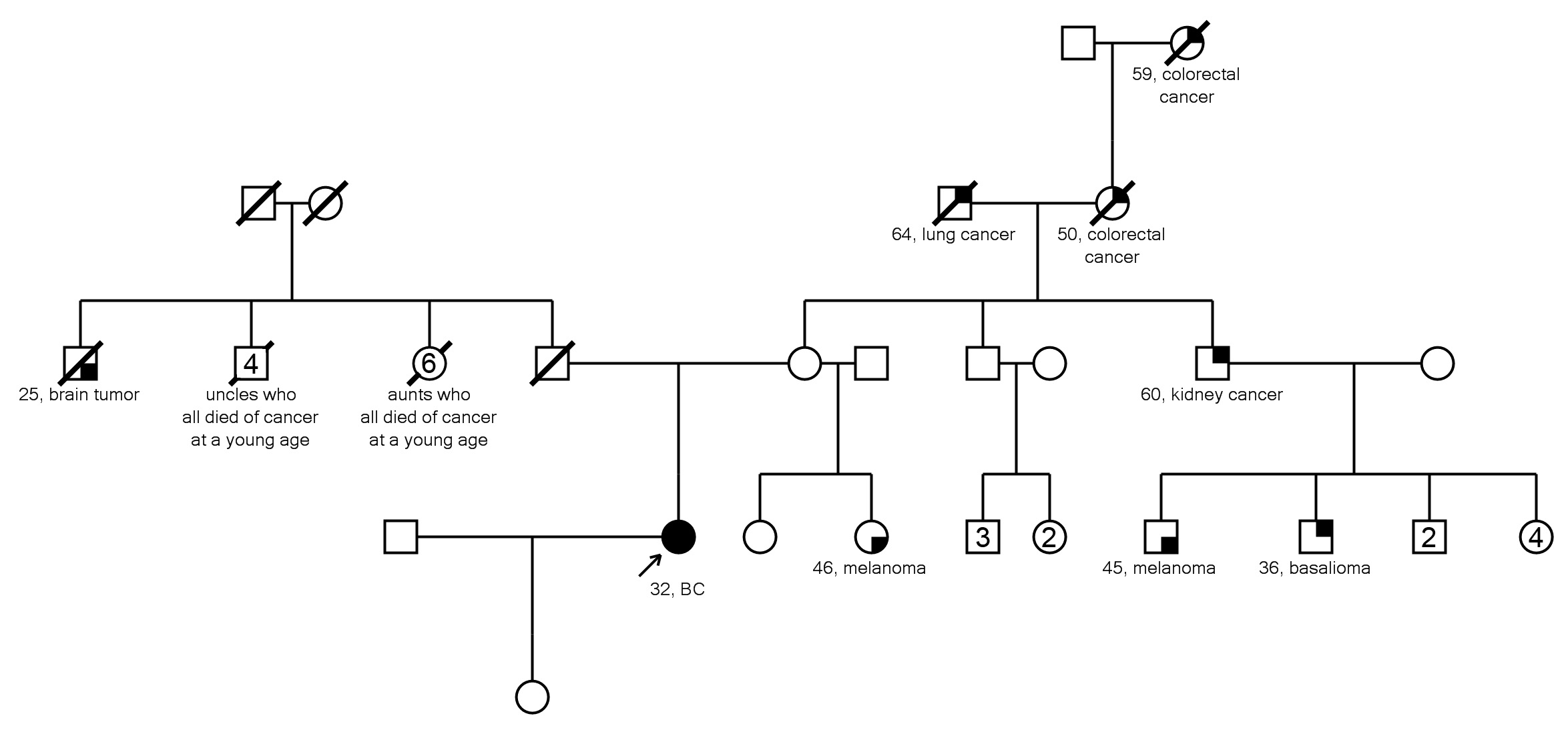


1. Patient #78, *WRN* exon 15-16 del


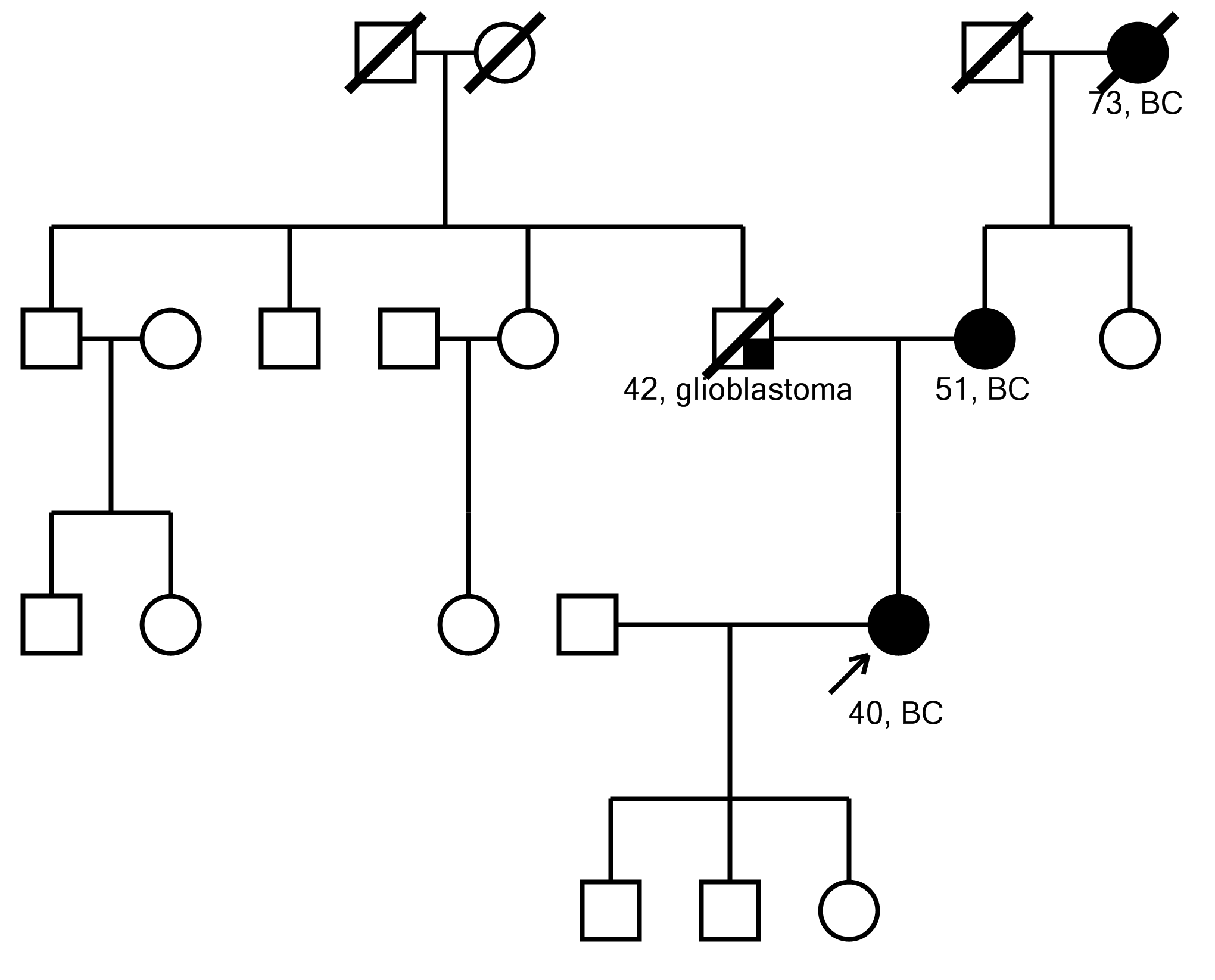


1. Patient #79, ATM:p.(Glu1978*)


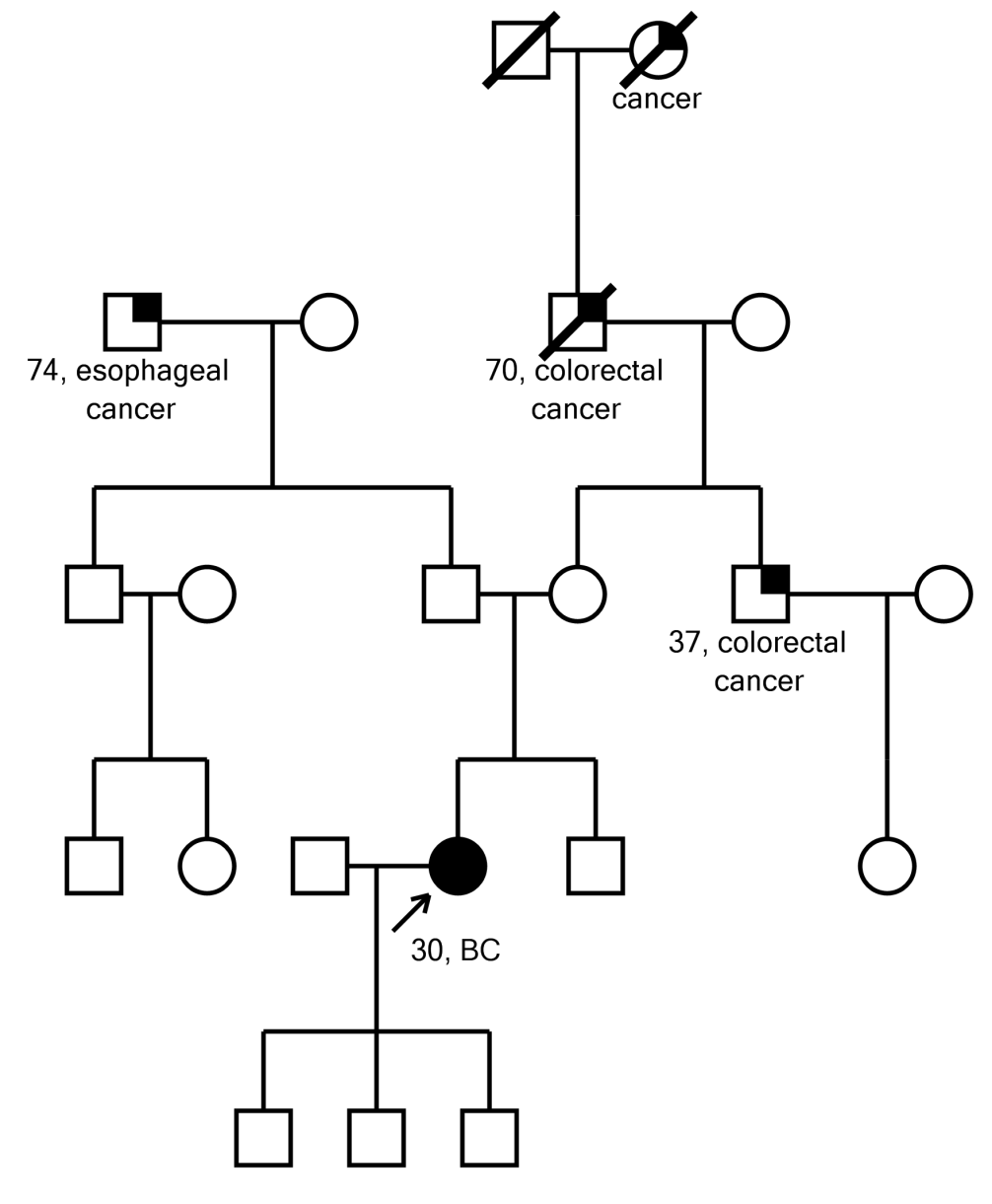


1. Patient #84, *PMS2* exon 3-8 del, FANCI:p.(Arg1285*)


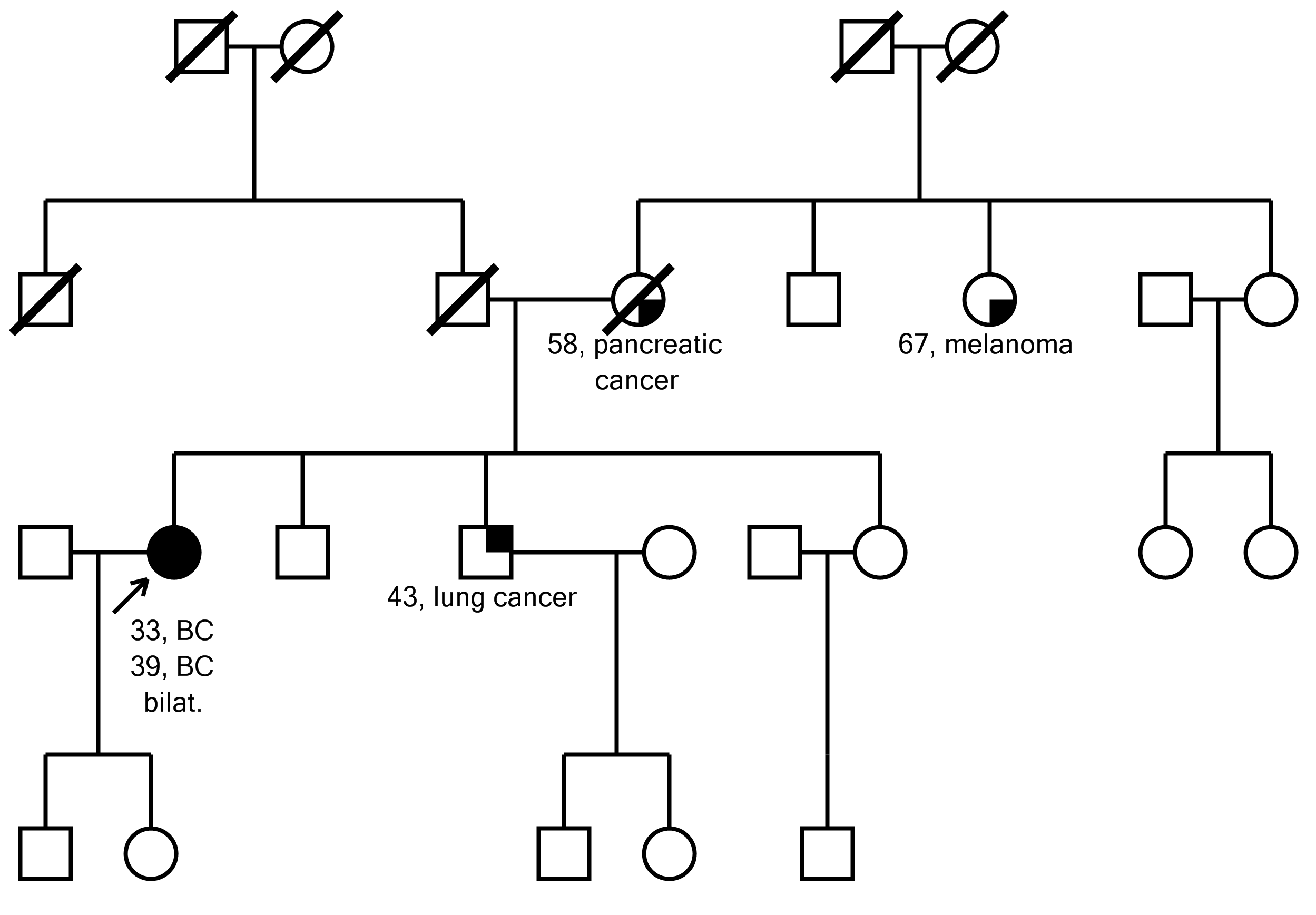


1. Patient #96, PALB2:p.(Gln60Argfs*7)


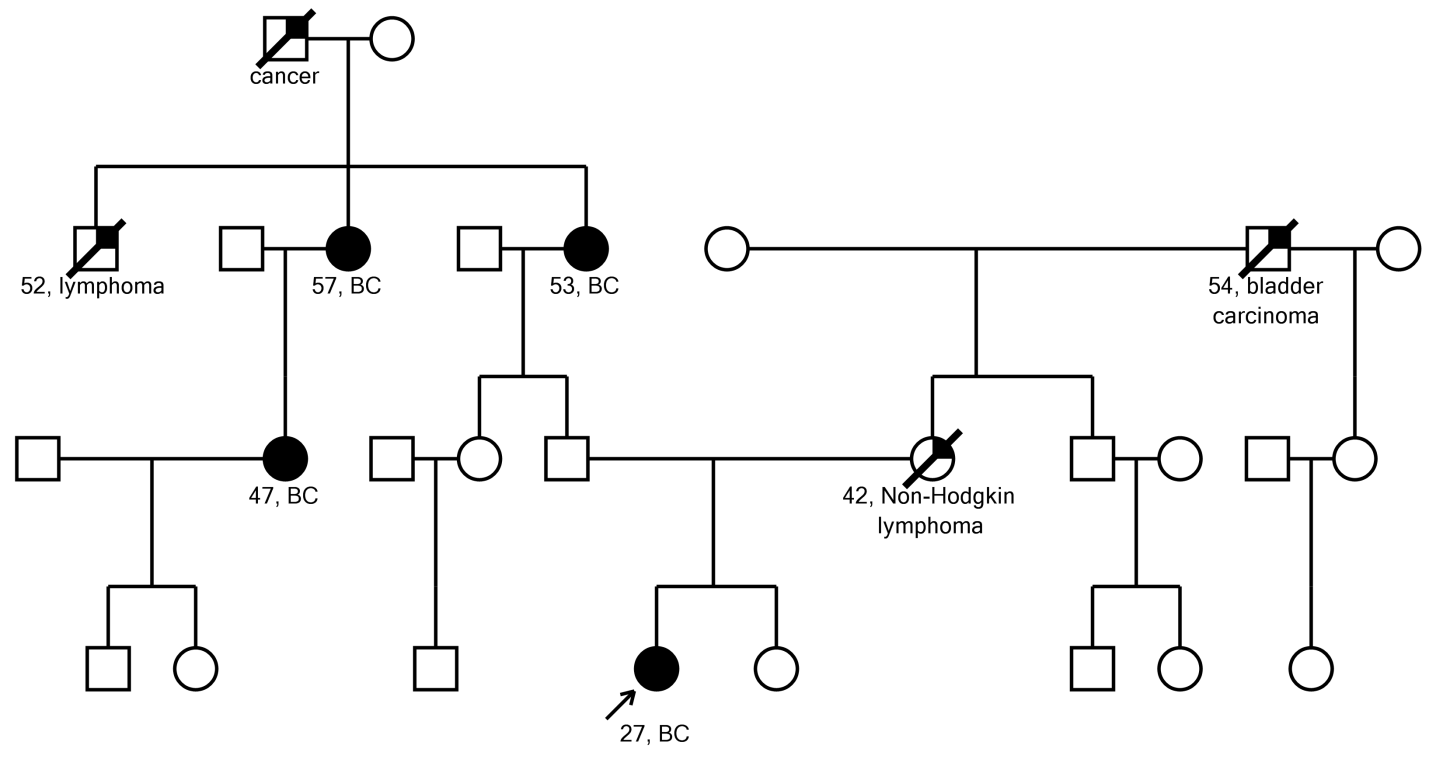


1. Patient #99, RECQL4:p.(Ala919Thr)
