# Supplementary material for: Breast cancer patients suggestive of Li-Fraumeni syndrome: mutational spectrum, candidate genes, and unexplained heredity
Source: Breast Cancer Res. 2018 Aug 7;20:87. doi: 10.1186/s13058-018-1011-1 (PMC6081832; doi:10.1186/s13058-018-1011-1)
Supplement: Supplementary file 4 — Total list of TP53 (NM_000546.5) variants detected via NGS-based sequencing (entire list of all detected TP53 variants in our collective). (DOCX 35 kb) [file 13058_2018_1011_MOESM4_ESM.docx]

**Additional File 4**

**Total list of *TP53* (NM_000546.5)** **variants detected via NGS-based sequencing**

| dbSNP | Index ID  Patient | Variant type | Exon/total no. of exons | cDNA change | Predicted  AA change | Total allele frequency,  gnomAD; population with highest AF* | homozygous/heterozygous |
| --- | --- | --- | --- | --- | --- | --- | --- |
| rs1042522 | 1 | missense | 4/11 | c.215C>G | p.(Pro72Arg) | 0.6631;  NFE: 0.7366 | hom |
| rs1042522 | 2 | missense | 4/11 | c.215C>G | p.(Pro72Arg) | 0.6631;  NFE: 0.7366 | hom |
| rs1042522 | 3 | missense | 4/11 | c.215C>G | p.(Pro72Arg) | 0.6631;  NFE: 0.7366 | het |
| rs1042522 | 4 | missense | 4/11 | c.215C>G | p.(Pro72Arg) | 0.6631;  NFE: 0.7366 | het |
| rs1042522 | 5 | missense | 4/11 | c.215C>G | p.(Pro72Arg) | 0.6631;  NFE: 0.7366 | het |
| rs1042522 | 6 | missense | 4/11 | c.215C>G | p.(Pro72Arg) | 0.6631;  NFE: 0.7366 | hom |
| rs1042522 | 7 | missense | 4/11 | c.215C>G | p.(Pro72Arg) | 0.6631;  NFE: 0.7366 | het |
| rs1042522 | 8 | missense | 4/11 | c.215C>G | p.(Pro72Arg) | 0.6631;  NFE: 0.7366 | het |
| rs1042522 | 9 | missense | 4/11 | c.215C>G | p.(Pro72Arg) | 0.6631;  NFE: 0.7366 | hom |
| rs1042522 | 10 | missense | 4/11 | c.215C>G | p.(Pro72Arg) | 0.6631;  NFE: 0.7366 | het |
| rs1042522 | 11 | missense | 4/11 | c.215C>G | p.(Pro72Arg) | 0.6631;  NFE: 0.7366 | het |
| rs1042522 | 12 | missense | 4/11 | c.215C>G | p.(Pro72Arg) | 0.6631;  NFE: 0.7366 | hom |
| rs1042522 | 13 | missense | 4/11 | c.215C>G | p.(Pro72Arg) | 0.6631;  NFE: 0.7366 | het |
| rs1042522 | 14 | missense | 4/11 | c.215C>G | p.(Pro72Arg) | 0.6631;  NFE: 0.7366 | hom |
| rs1042522 | 15 | missense | 4/11 | c.215C>G | p.(Pro72Arg) | 0.6631;  NFE: 0.7366 | hom |
| rs1042522 | 17 | missense | 4/11 | c.215C>G | p.(Pro72Arg) | 0.6631;  NFE: 0.7366 | hom |
| rs1042522 | 18 | missense | 4/11 | c.215C>G | p.(Pro72Arg) | 0.6631;  NFE: 0.7366 | hom |
| rs1042522 | 19 | missense | 4/11 | c.215C>G | p.(Pro72Arg) | 0.6631;  NFE: 0.7366 | het |
| rs1042522 | 20 | missense | 4/11 | c.215C>G | p.(Pro72Arg) | 0.6631;  NFE: 0.7366 | hom |
| rs1042522 | 22 | missense | 4/11 | c.215C>G | p.(Pro72Arg) | 0.6631;  NFE: 0.7366 | het |
| rs1042522 | 23 | missense | 4/11 | c.215C>G | p.(Pro72Arg) | 0.6631;  NFE: 0.7366 | hom |
| rs1042522 | 24 | missense | 4/11 | c.215C>G | p.(Pro72Arg) | 0.6631;  NFE: 0.7366 | hom |
| rs1042522 | 25 | missense | 4/11 | c.215C>G | p.(Pro72Arg) | 0.6631;  NFE: 0.7366 | het |
| rs1042522 | 26 | missense | 4/11 | c.215C>G | p.(Pro72Arg) | 0.6631;  NFE: 0.7366 | het |
| rs1042522 | 27 | missense | 4/11 | c.215C>G | p.(Pro72Arg) | 0.6631;  NFE: 0.7366 | hom |
| rs1042522 | 28 | missense | 4/11 | c.215C>G | p.(Pro72Arg) | 0.6631;  NFE: 0.7366 | het |
| rs1042522 | 29 | missense | 4/11 | c.215C>G | p.(Pro72Arg) | 0.6631;  NFE: 0.7366 | hom |
| rs1042522 | 30 | missense | 4/11 | c.215C>G | p.(Pro72Arg) | 0.6631;  NFE: 0.7366 | hom |
| rs1042522 | 31 | missense | 4/11 | c.215C>G | p.(Pro72Arg) | 0.6631;  NFE: 0.7366 | het |
| rs1042522 | 32 | missense | 4/11 | c.215C>G | p.(Pro72Arg) | 0.6631;  NFE: 0.7366 | het |
| rs1042522 | 33 | missense | 4/11 | c.215C>G | p.(Pro72Arg) | 0.6631;  NFE: 0.7366 | het |
| rs1042522 | 34 | missense | 4/11 | c.215C>G | p.(Pro72Arg) | 0.6631;  NFE: 0.7366 | hom |
| rs1042522 | 35 | missense | 4/11 | c.215C>G | p.(Pro72Arg) | 0.6631;  NFE: 0.7366 | hom |
| rs1042522 | 36 | missense | 4/11 | c.215C>G | p.(Pro72Arg) | 0.6631;  NFE: 0.7366 | hom |
| rs1042522 | 37 | missense | 4/11 | c.215C>G | p.(Pro72Arg) | 0.6631;  NFE: 0.7366 | het |
| rs1042522 | 38 | missense | 4/11 | c.215C>G | p.(Pro72Arg) | 0.6631;  NFE: 0.7366 | het |
| rs1042522 | 39 | missense | 4/11 | c.215C>G | p.(Pro72Arg) | 0.6631;  NFE: 0.7366 | hom |
| rs1042522 | 42 | missense | 4/11 | c.215C>G | p.(Pro72Arg) | 0.6631;  NFE: 0.7366 | hom |
| rs1042522 | 44 | missense | 4/11 | c.215C>G | p.(Pro72Arg) | 0.6631;  NFE: 0.7366 | hom |
| rs1042522 | 45 | missense | 4/11 | c.215C>G | p.(Pro72Arg) | 0.6631;  NFE: 0.7366 | hom |
| rs1042522 | 46 | missense | 4/11 | c.215C>G | p.(Pro72Arg) | 0.6631;  NFE: 0.7366 | hom |
| rs1042522 | 47 | missense | 4/11 | c.215C>G | p.(Pro72Arg) | 0.6631;  NFE: 0.7366 | het |
| rs1042522 | 48 | missense | 4/11 | c.215C>G | p.(Pro72Arg) | 0.6631;  NFE: 0.7366 | hom |
| rs1042522 | 50 | missense | 4/11 | c.215C>G | p.(Pro72Arg) | 0.6631;  NFE: 0.7366 | het |
| rs1042522 | 51 | missense | 4/11 | c.215C>G | p.(Pro72Arg) | 0.6631;  NFE: 0.7366 | het |
| rs1042522 | 52 | missense | 4/11 | c.215C>G | p.(Pro72Arg) | 0.6631;  NFE: 0.7366 | het |
| rs1042522 | 53 | missense | 4/11 | c.215C>G | p.(Pro72Arg) | 0.6631;  NFE: 0.7366 | het |
| rs1042522 | 54 | missense | 4/11 | c.215C>G | p.(Pro72Arg) | 0.6631;  NFE: 0.7366 | hom |
| rs1042522 | 55 | missense | 4/11 | c.215C>G | p.(Pro72Arg) | 0.6631;  NFE: 0.7366 | hom |
| rs1042522 | 56 | missense | 4/11 | c.215C>G | p.(Pro72Arg) | 0.6631;  NFE: 0.7366 | het |
| rs1042522 | 57 | missense | 4/11 | c.215C>G | p.(Pro72Arg) | 0.6631;  NFE: 0.7366 | hom |
| rs1042522 | 58 | missense | 4/11 | c.215C>G | p.(Pro72Arg) | 0.6631;  NFE: 0.7366 | hom |
| rs1042522 | 59 | missense | 4/11 | c.215C>G | p.(Pro72Arg) | 0.6631;  NFE: 0.7366 | het |
| rs1042522 | 60 | missense | 4/11 | c.215C>G | p.(Pro72Arg) | 0.6631;  NFE: 0.7366 | hom |
| rs1042522 | 61 | missense | 4/11 | c.215C>G | p.(Pro72Arg) | 0.6631;  NFE: 0.7366 | hom |
| rs1042522 | 62 | missense | 4/11 | c.215C>G | p.(Pro72Arg) | 0.6631;  NFE: 0.7366 | hom |
| rs1042522 | 63 | missense | 4/11 | c.215C>G | p.(Pro72Arg) | 0.6631;  NFE: 0.7366 | hom |
| rs1042522 | 64 | missense | 4/11 | c.215C>G | p.(Pro72Arg) | 0.6631;  NFE: 0.7366 | het |
| rs1042522 | 65 | missense | 4/11 | c.215C>G | p.(Pro72Arg) | 0.6631;  NFE: 0.7366 | hom |
| rs1042522 | 66 | missense | 4/11 | c.215C>G | p.(Pro72Arg) | 0.6631;  NFE: 0.7366 | hom |
| rs1042522 | 67 | missense | 4/11 | c.215C>G | p.(Pro72Arg) | 0.6631;  NFE: 0.7366 | het |
| rs1042522 | 68 | missense | 4/11 | c.215C>G | p.(Pro72Arg) | 0.6631;  NFE: 0.7366 | hom |
| rs1042522 | 69 | missense | 4/11 | c.215C>G | p.(Pro72Arg) | 0.6631;  NFE: 0.7366 | hom |
| rs1042522 | 70 | missense | 4/11 | c.215C>G | p.(Pro72Arg) | 0.6631;  NFE: 0.7366 | hom |
| rs1042522 | 71 | missense | 4/11 | c.215C>G | p.(Pro72Arg) | 0.6631;  NFE: 0.7366 | hom |
| rs1042522 | 72 | missense | 4/11 | c.215C>G | p.(Pro72Arg) | 0.6631;  NFE: 0.7366 | hom |
| rs1042522 | 73 | missense | 4/11 | c.215C>G | p.(Pro72Arg) | 0.6631;  NFE: 0.7366 | hom |
| rs1042522 | 75 | missense | 4/11 | c.215C>G | p.(Pro72Arg) | 0.6631;  NFE: 0.7366 | hom |
| rs1042522 | 76 | missense | 4/11 | c.215C>G | p.(Pro72Arg) | 0.6631;  NFE: 0.7366 | hom |
| rs1042522 | 77 | missense | 4/11 | c.215C>G | p.(Pro72Arg) | 0.6631;  NFE: 0.7366 | het |
| rs1042522 | 78 | missense | 4/11 | c.215C>G | p.(Pro72Arg) | 0.6631;  NFE: 0.7366 | het |
| rs1042522 | 79 | missense | 4/11 | c.215C>G | p.(Pro72Arg) | 0.6631;  NFE: 0.7366 | het |
| rs1042522 | 80 | missense | 4/11 | c.215C>G | p.(Pro72Arg) | 0.6631;  NFE: 0.7366 | hom |
| rs1042522 | 81 | missense | 4/11 | c.215C>G | p.(Pro72Arg) | 0.6631;  NFE: 0.7366 | hom |
| rs1042522 | 82 | missense | 4/11 | c.215C>G | p.(Pro72Arg) | 0.6631;  NFE: 0.7366 | hom |
| rs1042522 | 83 | missense | 4/11 | c.215C>G | p.(Pro72Arg) | 0.6631;  NFE: 0.7366 | hom |
|  |  |  |  |  |  |  |  |
| rs17880847 | 29 | intron |  | c.1100+30A>T |  | 0.009329;  AJ: 0.01835 | het |
| rs17880847 | 57 | intron |  | c.1100+30A>T |  | 0.009329;  AJ: 0.01835 | het |
| rs17880847 | 66 | intron |  | c.1100+30A>T |  | 0.009329;  AJ: 0.01835 | het |
|  |  |  |  |  |  |  |  |
| rs17883323 | 3 | intron |  | c.97-29C>A |  | 0.06676;  FE: 0.1075 | het |
| rs17883323 | 10 | intron |  | c.97-29C>A |  | 0.06676;  FE: 0.1075 | het |
| rs17883323 | 16 | intron |  | c.97-29C>A |  | 0.06676;  FE: 0.1075 | het |
| rs17883323 | 21 | intron |  | c.97-29C>A |  | 0.06676;  FE: 0.1075 | het |
| rs17883323 | 22 | intron |  | c.97-29C>A |  | 0.06676;  FE: 0.1075 | het |
| rs17883323 | 31 | intron |  | c.97-29C>A |  | 0.06676;  FE: 0.1075 | het |
| rs17883323 | 40 | intron |  | c.97-29C>A |  | 0.06676;  FE: 0.1075 | het |
| rs17883323 | 43 | intron |  | c.97-29C>A |  | 0.06676;  FE: 0.1075 | het |
| rs17883323 | 49 | intron |  | c.97-29C>A |  | 0.06676;  FE: 0.1075 | het |
| rs17883323 | 59 | intron |  | c.97-29C>A |  | 0.06676;  FE: 0.1075 | het |
| rs17883323 | 74 | intron |  | c.97-29C>A |  | 0.06676;  FE: 0.1075 | het |
|  |  |  |  |  |  |  |  |
| rs1800372 | 23 | synonymous | 6/11 | c.639A>G | p.Arg213Arg | 0.01229;  AJ: 0.03379 | het |

*NFE = European (Non-Finnish); FE = European (Finnish); A = African; L = Latino; EA = East Asian; SA = South Asian; AJ = Ashkenazi Jewish; O = Other
